# Pattern of blast injuries. A systematic review: Part 2 – Landmines, unexploded ordnance and terrorism

**DOI:** 10.1007/s00068-026-03241-1

**Published:** 2026-06-18

**Authors:** Anne Neubert, Catharina Gaeth, Max Seidelmann, Dan Bieler

**Affiliations:** 1 Department of Orthopedics and Traumatology, University Hospital and Medical Faculty, Heinrich-Heine-University Duesseldorf, Moorenstraße 5, 40225 Duesseldorf, Germany; 2 TraumaEvidence @ Germany Trauma Society, Berlin, Germany; 3 Department of Trauma Surgery and Orthopedics, Hand-, Reconstructive Surgery and Plastic Surgery, Plastic Surgery and Burn Medicine, German Armed Forces Central Hospital, Medical Campus Koblenz of University Medicine Mainz, Ruebenacher Str. 170, 56072 Koblenz, Germany

**Keywords:** Blast injury, Explosion, Injury pattern, Extremity injuries, Amputation, War, Terror, Landmine, Conflicts

## Abstract

**Purpose:**

This systematic review analyzes the patterns and distribution of blast injuries from landmine explosions, UXOs and terror attacks including suicide bombing, in civilians and military personnel.

**Methods:**

A search was conducted in two databases on 26. March 2025 for observational studies reporting blast injuries in civilian and military populations of all ages. Studies were clustered by explosion typ. A narrative data synthesis was performed. The JBI tool for case series was used. PROSPERO: CRD42023391736.

**Results:**

126 studies were included in the overall systematic review. 45 studies on *terror & suicide bombings* and 12 studies on *landmines &* UXOs are analyzed in the present paper. Studies on landmines from 1980 to 2006 included 13,270 individuals (age: 24.0 years (Range 8.2 years – 32.2 years); mainly male (88.9%)) in post-conflict low- and middle-income countries. Lower extremity injuries including amputations were most frequent, followed by eye injuries. Studies on terrorist attacks with a total of 12,518 mainly civilian victims (average age: 33,1 years; 61.6% males) reported on incidents in post-conflict regions and high-income cities mainly from the early 2000s. The injury pattern was multidimensional, with extremity injuries most frequent (28%-30%), including fractures, lacerations, and traumatic amputations (18%-20%).

**Conclusion:**

This Systematic Review Part 2 on landmines and terror attacks found that extremity injuries, including traumatic amputations, are the most frequent and dominate injury. However, the injury pattern varies depending on the explosion mechanism. Landmines primarily cause lower extremity injuries, as well as eye injuries. Terror and suicide explosions exhibit multidimensional injury pattern depending on the distance and location of the explosion.

**Supplementary Information:**

The online version contains supplementary material available at 10.1007/s00068-026-03241-1.

## Introduction

Blast injuries are complex and multifaceted, encompassing traumatic amputations, shrapnel injuries, and internal injuries such as blast lung. Notably, they are not confined to warfare. They are increasingly common in the civilian areas due to terrorist attacks and suicide bombings, as well as in post-conflict regions contaminated by landmines and war remnants.

Terrorism is defined as the deliberate intention to cause fear and violence through psychological and physical warfare to harm, injure, or kill as many (civilian) victims as possible [1–6]. This is often done to express an opinion, or intimidate others to convey politically, religiously or ideologically motivated goals [4, 5]. Statistics show that terrorism caused 21,596 deaths in 2023, underscoring the relevance of this issue [7]. A common modus operandi is bombing, with the intent of causing mass casualties, system disruptions, and resource consumption, especially in the medical care sector [1, 4, 8–10]. Consequently, terrorist attacks often occur in urban environments, targeting crowded places such as train stations (for example, Madrid) and buses (for example, Jerusalem) [11].

In contrast, landmines are explosive devices used in conflicts to target individuals or vehicles. These are concealed weapons that remain buried in the ground [12]. They are commonly used to defend land borders, such as those between Pakistan and India. Landmines are designed to cause severe injuries with the intent of causing morbidity rather than mortality, thereby demoralizing troops while imposing economic and rehabilitation burdens [13, 14]. In addition to landmines, unexploded ordnances of war (UXOs), including grenades, aerial bombs, and mortars, pose significant threats to life and limb. These explosive devices remain in the ground long after conflicts end and are particularly hazardous to civilians because of their indiscriminate nature [12–17]. In 2023, at least 5,757 people were killed or injured by war remnants in contaminated areas [17]. Consequently, landmines and UXOs present humanitarian challenges, even after conflicts have concluded.

The large number of victims suggests the need to raise awareness and improve understanding of blast injuries and the unique injury patterns caused by landmines and various acts of terrorism. This knowledge is essential for enhancing patient care, outcomes, and survival by adapting training on specific diagnostics and treatment of blast injuries, particularly in the civilian healthcare sector, as blast injuries resulting from terrorist attacks can occur unpredictably.

Despite studies reporting on different terrorist events and landmine injuries, primarily from retrospective hospital reports and registries, there is a lack of comprehensive analysis of injury patterns based on specific blast mechanisms. Rarely do studies address the specific injuries in landmine victims and terrorist attacks. Although blast injuries are typically characterized using the blast injury classification system, it only emphasizes the mechanisms of explosions and their pathophysiological impacts. This system categorizes blast injuries into primary, secondary, tertiary, quaternary, or quinary, as detailed in general literature [2, 4, 18]. However, there is a lack of detailed knowledge and awareness of specific types of blast injuries, especially in non-conflict regions such as Germany, where such injuries are infrequently encountered.

This systematic review aimed to analyze the specific injury patterns and distribution of blast injuries caused by landmine or UXO explosions, and terror attacks, including suicide bombings harming civilians and military personnel. Here Part 2 on landmine or UXO as well as terror attacks and suicide bombing is presented.

## Methods

The protocol for the systematic review was registered with PROSPERO (registration number: CRD42023391736). The systematic review reports according to the “Preferred Reporting Items for Systematic reviews and Meta-Analyses” (PRISMA) guidelines (checklist provided in Additional File 1) [19]. For a complete overview of the methods, please refer to Part 1 of this systematic review: “Pattern of blast injuries Part 1 – Improvised explosive devices, multiple & unspecified mechanisms”. Only an overview of the key methods used are provided here.

A systematic search on MEDLINE via PubMed and Scopus was conducted on 26. March 2025. We included all observational studies that reported injuries classified as blast injuries, encompassing primary, secondary, tertiary, quaternary, and quinary injuries in civilians and military personnel of all age groups. Selection, data extraction, and assessment of reporting quality were performed by two independent reviewers. The JBI tool for case series was used to assess the reporting quality of the included studies [20].

All studies were clustered into one of the following groups: (1) improvised explosive devices (IED) (2), multiple or not specified explosive devices (3), landmines or UXOs (4) terror attacks & suicide bombings. All four categories were analyzed separately. In Part 1, the injuries caused by IEDs, multiple, or not specific explosive devices are presented in detail (Neubert et al. in prep – Part 1). Part 2 encompasses (3) landmines or UXOs or (4) terror attacks and suicide bombings. We clustered studies under the rubric of landmines and UXOs if the authors explicitly classified the explosion as such in the methods section of the included studies. Furthermore, studies on terror attacks and suicide bombings were sorted in the rubric if the authors explicitly classified the explosion as such in the methods of the study. Hence, it cannot be ruled out that some of the studies could also belong to other rubrics, such as IED, as IEDs are also used in terror attacks or as mines. Therefore, this part of the systematic review was separated from Part 1. Studies on landmines or UXOs focus particularly on post-conflict or conflict-ridden countries. The studies included reported explosions mainly causing injuries in civilians. However, landmines can be used both offensively and defensively. Whereas IEDs and multiple explosive devices are especially used in asymmetric conflicts (military versus non-state armed group(s)), such as the conflicts in Iraq and Afghanistan [21]. Studies on terror attacks (and suicide bombings) are widespread and cause harm mainly to civilians. Terror attacks are common in industrialized countries, whereas suicide bombings are more common in post-conflict or conflict-ridden countries [22].

Where possible, injuries were assigned to one of the body areas, as described in Table 1. Only amputations were reported and not differentiated as minor or major. Injury pattern were organized by body region using the Abbreviated Injury Score (AIS) [23, 24].

**Table 1:** Categorization of injuries

General categories	Included injuries / regions
Head/neck	• Brain and head excluding facial region • Neck injuries including/excluding cervical spine
Face	• Eyes, ears, facial region (e.g. jaw)
Abdomen	• Excluding spine (if detail was available)
Thorax	• Including bone and internal organ damage • Excluding spine (if detail was available)
Extremities	• Including fractures and amputations as soft tissues injuries were only rarely reported in detail • Often includes clavicular and scapular injuries • Excluding soft tissues injuries (if detail was available)
External incl. burns	• Including burns (reporting mostly on general external injuries, without sufficient details to differentiate burns from other external injuries)
If details are available	
Pelvis	If detailed information was available • Includes all reported fractures However, pelvic injuries could have been reported as part of extremity or abdominal injuries.
Spine	If detailed information was available • Includes all reported fractures However, spine injuries could have been reported as part of head/neck, thorax or abdominal injuries.
Urogenital	If detailed information was available • All reported injuries that involve the urogenital tracts However, urogenital injuries could have been reported as part of abdominal injuries.

Due to the high degree of heterogeneity of different circumstances, populations, and injury mechanisms, no meta-analyses have been conducted. Where possible, descriptive statistics, graphs, and tables were used to illustrate the identified patterns of injury. Many studies were retrospective register data analyses that investigated overlapping study populations, which hindered more profound insights and descriptive statistical analyses. Due to the overlap in study populations, especially in relation to register studies and the missing reporting for many body regions, only studies with a large population and the most detailed reporting were used for graphs. Other studies from the same register and timeframe were disregarded as they investigate overlapping populations or subgroups of the same population. The procedure is reported in the graphs and sections, if used.

## Results

**Fig. 1:**
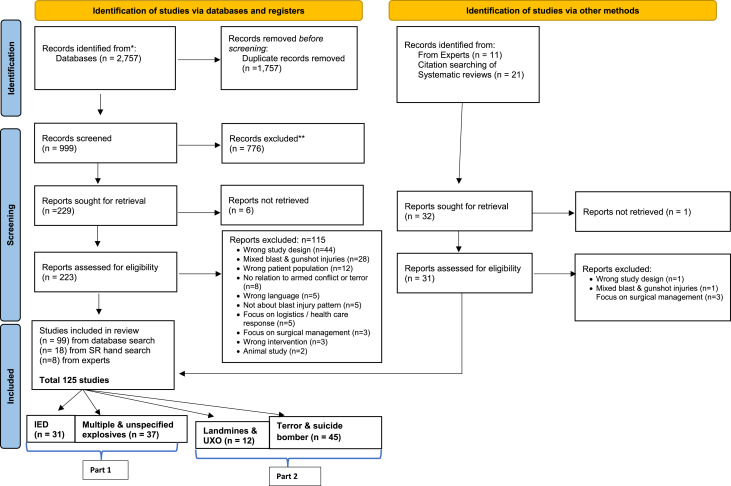
PRISMA Flowchart

The database search revealed a total of 2,756 hits after title/abstract, and full-text screening, including studies from a manual search. A total of 125 studies were included in the systematic review. Of these, twelve studies on landmines and UXOs as well as 45 studies on terror, including suicide bombings, are reported here. The PRISMA flowchart illustrating the screening process is shown in Fig. 1. An overview of the excluded studies is provided in “Pattern of blast injuries. Part 1” (Neubert et al., in prep).

### Results on landmines & UXO

Twelve studies reporting injuries caused by landmines and UXOs were included. The total study population consisted of 13,270 individuals who were injured between 1980 and 2006. The timeframe of three studies was unknown [13, 14, 25–34]. The studies reported landmine injuries in Iran (*n* = 3), Afghanistan (*n* = 3), Sri Lanka (*n* = 2), and one each in Pakistan, India, Chechnya, and an unknown country. In six studies, the population under investigation was military personnel and civilians [14, 25–29], while in five, it was civil population [13, 24, 26–28], and in one, only military personnel [31]. Most studies obtained data from hospital records or registers, such as the International Committee of the Red Cross (ICRC) [26, 27], Information Management System for Mine Action [28], or a register for patient acquisition [33]. Only one study was prospective [14]. In most studies, children and adults were reported together. One study reported exclusively on injured children [33], and two other studies on injured adults [13, 31]. The mean age across studies was 24.0 years (range: 8.2– 32.2 years). Of the population, 88.9% were men (missing sex distribution in one study [29]). Further details are provided in Table 2.

**Table 2:** Characteristics of included studies – landmines & UXO

Study ID	*N*	Age in years	Sex^b^	Country	Time frame	Context	Focus of the study	JBI
Afshar 2007 [25]	156	65% 15–35	95%	Iran	1998–2004	C & M	General injury pattern	7
Bilukha 2003 [27]	1,636	46% < 16	92%	Afghanistan	March 2001 -June 2002	C & M	General injury pattern	10
Bilukha 2007 [28]	3,021	majority 15–24	81%	Chechnya	1994–2005	C & M	Amputations	10
Bilukha 2008 [26]	5,471	47.2% (0–17)	91%	Afghanistan	January 2002 -December 2006	C & M	Amputations	9
Coupland 1991 [29]	757	-	-	Ø	1 year	C & M	General injury pattern	7
Damodar 2015 [30]	54	range 10–60	87%	Vijaywada, India	-	C	General injury pattern	5
Goonetilleke 1995 [31]	191	-	100%	Palaly & Colombo, Sri Lanka	June 1990 -August 1992	M	Amputations	8
Khan 2002 [14]	28	Ø 17.75 (13-55y)	100%	Kashmir Pakistan	-	C & M	Lower limb injuries	10
Meade 2000 [32]	328	Ø 32.2 (6 month − 88)	81%	Jaffna, Sri Lanka	May 1, 1996 -December 31, 1997	C	General injury pattern	10
Mousavi 2015 [33]	78	Ø 8.2 (± 3.12)	86%	Iran	1980–1988	C	General injury pattern	10
Muzaffar 2000 [13]	51	Ø 29 (range 19–56)	100%	Afghanistan	November 1992 - January 1996	C	Eye injuries	10
Soroush 2008 [34]	1,499	Ø 23 (± 13)	92%	Iran	August 20, 1988 - March 20, 2003	C	General injury pattern	10

Legend: ^a^ context: C=civilian, M=military, C&M= mixed population of civilians and military personnel; ^**b**^ Sex: percentage of male victims

ICRC = International Committee of the Red Cross; JBI= Joanne Briggs Institute - risk of bias assessment; study ID = study identification; Ø = mean; - = not reported

### Issues with reporting & risk of bias

The risk of bias assessment showed an average of 8.8 out of 10 possible points (Range 5–10). An overview of the risk of bias assessment is provided in Additional File 2. Among the studies included, some issues regarding reporting were detected. Several studies have reported injuries according to the classification by Coupland and Korver (1991), which is based on the experiences of the International Committee of the Red Cross [29]. The classification of injury patterns is contingent upon the casualty’s proximity to the detonation of the landmine or UXO for example *“stood on a mine”.* It delineated three injury patterns: (1) Standing on a buried landmine, resulting in amputation of the leg either below or above the knee, often with damage to the contralateral leg; (2) Pertains to triggering a mine, such as by a tripwire, leading to a diffuse injury pattern, including potential injuries to the abdomen and chest from fragments; (3) Occurs during mine handling when an individual picks up the mine, often resulting in fatal injuries or associated with upper limb amputations and severe facial injuries [25–27, 29, 33]. Studies that employed this classification often only reported the type of pattern but not the injuries in detail. Injuries that do not fit the pattern are often left unreported or unexplained for example, Mousavi (2015) reported the remaining injuries as “*other injuries*” [33]. Furthermore, amputations were often only reported as *“number of persons with an amputation”.*

### General injury pattern

The distribution of injuries shown in the heatmap in Table 3 demonstrates the predominance of extremity and eye injuries in this group of explosions. This is also reflected in the classification of landmine injuries by Coupland 1991 [29], as explained above. Only one study reported thoracic injuries [25]. However, as explained, the risk of underreporting injuries is high in this group of explosions. Extremity injuries are consistently the most commonly (≥ 70%) affected body regions. Facial and abdominal injuries occur notably more frequently in individual studies (for example, Mousavi 2015, Goonetilleke 1995), which may indicate different circumstances (e.g., civilian vs. military, type of mine, proximity to the explosion). Hence, head, thorax, and facial injuries are reported less frequently.

Some of the included studies offered a more detailed insight into extremity injuries [25, 30, 31, 33, 34]. The most common were lower extremity injuries, and the lower extremity was most often affected by amputations. Amputations were present throughout the studies included but showed variability in frequency.

**Table 3:** Heatmap landmines & UXO

Population	Study- ID	Injury severity	Total (*n*)	Head	Face	Thorax	Abdomen	Extremity
**Reported according to number of injured persons**								
C & M	Afshar 2007 [25]	Unknown	156	-	22%	9%	-	82%
Military	Goonetilleke 1995 [31]		191	5%		-	-	80%
Civil	Mousavi 2015 [33]		78	-	58%	-	-	53%
**Reported according to number of injuries**								
Civil	Damodar 2015 [30]	Unknown	142	1.5%	8%	-	54%	-
	Soroush 2008 [34]	Unknown	4,153	8%	3%	1%	2%	84%

Legend: color scheme < 10%; 10–29%; 30–49%; 50–69%; ≥70%; – = not reported; JTTR = Joint Theatre Trauma Registry

### Injury pattern – focus on specific injuries

Five studies focused on specific body parts. One study investigated eye injuries in Afghanistan [13]. Four studies focused on amputations [14, 26, 28, 31]. Further details can be retrieved from Additional File 3 –Additional information on landmines and UXOs.

### Results on terror attacks including suicide bombings

45 studies reported injuries caused by terror attacks or suicide bombings with a total of 12,518 victims [1, 35–78]. The reported terror attacks or suicide bombing events happened between 1969 and 2021 with most of them occurring between 2002 and 2004 [1, 37, 40, 43, 45, 53, 57, 59, 64, 67, 71]. 21 studies reported multiple events in different time frames, while the other 24 focused on single events on a specific date. Regarding the geographical distribution most studies are from Israel [1, 35–37, 43, 45, 47–51, 63–65, 67, 72] followed by six studies from the United Kingdom (UK) [41, 46, 58, 70, 73, 76]. Three studies each were from Spain [53, 59, 71], Saudi-Arabia [69, 77] and the United States of America (USA) [52, 54, 61]. Two studies were each from Italy [39, 42], Northern Ireland [44, 60], Pakistan [57, 74], and Turkey [56, 75]. The remaining studies were from multiple countries in Southeast Asia, Africa, South America, Europe, and the Middle East (Table 4).

These studies mostly used retrospective hospital data. Almogy 2005 and Kluger 2005 combined hospital reports with data from the Israel National Trauma Register (ITR) [35, 48]. Other studies used only the ITR [43, 45, 64] or surveys as data sources [57, 77]. Many of the included studies investigated civilians, whereas three investigated a mixed population of civilians and military [67, 74, 77]. Three other studies investigated only military personnel [65, 66, 69]. Predominantly, adults were studied. From the 19 studies that reported the mean age, the average age was 33,1 years. Only one study reported exclusively on children [61], and one reported on children and adults [78]. Five studies did not provide any information regarding the age of the included population [72–76]. 61.6% of victims were males (in 26 included studies with sufficient data). Most of the included studies reported a higher number of male victims except for three studies [46, 52, 62] that analyzed terror incidents that occurred in industrialized countries, such as the UK, which might explain the higher number of female victims.

### Issues with reporting & risk of bias

The risk of bias was low for most studies, with an average study quality of 9.2 out of 10 points (Range 6–10). In 39% of the included studies, demographic information of patients was missing. An overview of the risk of bias assessment is provided in Additional File 2.

For most studies in this rubric, the number of persons injured per event is unclear, as several people might only be mildly injured (self-medication or use of outpatient facilities) and did not visit the hospital. Depending on the size of the event, patients might been brought to several hospitals. The severity of the event and the actual pattern of injury among all injured individuals are unknown. In relation, most studies do not provide insight into explosive devices and location (confined space or open space), which hinders a more profound analysis.

Among the studies, a large heterogeneity in reporting of external injuries was discovered. Very mild injuries, such as superficial lacerations, were reported along with larger wounds. This complicated the assessment of the severity of the event. Furthermore, the extent to which burns are a significant issue in terror attacks remains unknown. Several studies have reported burn injuries, but with a larger variation in detail. However, burns seem to be a more significant issue in terror attacks than for other explosive mechanisms [40, 41, 63].

**Table 4:** Characteristics of included studies - terror including suicide bombings

Study ID	*n*	Age in years	Sex ^b^	Country	Time frame	Context ^a^	Focus of the study	JBI
Ahmad 2024 [78]	129	range 4–65	-	Nigeria	January 2009 – December 2021	-	Maxillofacial injuries	6
Almogy 2005 [35]	951	Mdn 38 (range 0,3–85)	-	Israel	1994–1997	C	General injury pattern	9
Arslan2022 [36]	1,073	> 60 = 269 (25%)	70%	Israel	May 2014 - April 2021	C	General injury pattern	10
Bala 2008 [1]	21	Mdn 21 (IQR 17–28)	57%	Israel	October 2000 - December 2005	C	Abdominal injuries	10
Bala 2010 [37]	55	Mdn 28 (IQR 20–35)	52%	Israel	October 2000 – December 2005	C	Chest injuries	10
Biancolini 1999 [38]	18	Ø 34 (± 18)	-	Argentina	July 18, 1994	C	General injury pattern	10
Brismar 1982 [39]	107	Ø 27	62%	Italy	August 02, 1980	C	General injury pattern	9
Chim 2007 [40]	31	Mdn 32 (range 13–56)	55%	Southeast Asia	2002–2005	C	Burn injuries	10
Chukwu-Lobelu 2017 [41]	52	-	-	UK	July 7, 2005	C	Burn injuries	9
Franceschetti 2021 [42]	22	-	-	Italy	December 1969 - July 1993	C	General injury pattern of fatalities	9
Golan 2014 [43]	262	-	-	Israel	November 2000 & August 2004	C	General injury pattern	9
Hadden 1978 [44]	1,532	-	40%	North Ireland	1969–1972	C	General injury pattern	9
Heldenberg 2016 [45]	1,261	majority between 15–29	69%	Israel	September 2000– December 2005	C	Vascular trauma	9
Johnstone 1993 [46]	30	Ø 29.1 (range 12–56)	47%	UK	February 18, 1993	C	General injury pattern	10
Katz 1988 [47]	29	-	-	Israel	-	C	General injury pattern	9
Kluger 2005 [48]	91	Ø 65 (± 27,3)	-	Israel	2002	C	Secondary ballistic injuries	9
Kluger 2007 [49]	4	Ø 30.2 (range 24–38)	100%	Israel	April 30, 2003	C	Quinary pattern of blast injury	7
Leibovici 1996 [51]	297	range 3–82	54%	Israel	1996	C	General injury pattern	10
Leibovici 1999 [50]	193	Ø 31.8 (range 8–81)	51%	Israel	1994–1996	C	Ear injuries	10
Mallonee 1996 [52]	759	Mdn 39 (84% 20–64)	43%	USA	April 19, 1995	C	General injury pattern	10
Martí 2006 [53]	36	Ø 37 (range 16–57)	56%	Spain	March 11, 2004	C	General injury pattern	10
Mines 2000 [54]	55	Ø 35 (range 1–65)	49%	USA	April 19, 1995	C	Eye injuries	10
Odhiambo 2002 [55]	290	Ø 31.6 (18–66)	65%	Kenia	1998	C	Maxillofacial incl. eye injuries	10
Parlak 2020 [56]	77	Ø 34.6 (range 15–75)	74%	Turkey	October 10, 2015	C	General injury pattern	10
Pasquier n.d. [57]	12	-	-	Pakistan	May 08, 2002	C	General injury pattern	9
Patel 2012 [58]	222	-	-	UK	July 7, 2005	C	Amputations	9
Peral Gutierrez de Ceballos 2005 [59]	243	Ø 32 (range 14–63)	59%	Spain	March 11, 2004	C	General injury pattern	10
Pyper 1983 [60]	339	-	-	Northern Ireland	1972–1980	C	General injury pattern	9
Quintana 1997 [61]	26	0.3 − 15	65%	USA	April 19, 1995	C	General injury pattern	10
Rignault 1989 [62]	205	Ø 34.5 (range > 0–89)	48%	France	1985–1986	C	General injury pattern	10
Rosenberg 1982 [63]	12	range 6–71	-	Israel	1975–1976	C	Burn injuries	8
Rozenfeld 2019 [64]	1,025	range 0–60	60%	Israel	January 1997 - December 2016	C	General injury pattern	9
Schwartz 2009 [65]	76	-	-	Israel	September 11, 2007	M	General injury pattern	9
Scott 1986 [66]	346	-	-	Lebanon	October 23, 1983	M	General injury pattern	6
Sheffy 2006 [67]	208	Ø 28 (± 14.6)	60%	Israel	September 29, 2000 - December 31, 2004	C&M	General injury pattern	10
Tahtabasi 2021 [68]	63	Ø 28.6 (± 10.2)	62%	Somalia	December 28, 2019	C	General injury pattern	10
Thach 2000 [69]	3	Ø 33 (22–40)	67%	Saudi Arabia	June 25, 1996	M	Ocular injuries	10
Thompson 2004 [77]	420	Ø 32 (± 5.9)	-	Saudi Arabia	June 25, 1996	C&M	General injury pattern	9
Tucker 1975 [70]	37	-	-	UK	1975	C	General injury pattern	8
Turégano-Fuentes 2008 [71]	512	-	-	Spain	Mach 11, 2004	C	General injury pattern	9
Waterworth 1975 [76]	21	-	-	UK	November 21, 1974	C	General injury pattern of fatalities	9
Weil 2007 [72]	44	Ø 20 (± 12.8)	-	Israel	2000–2003	C	Long bone fractures	9
Wong 2006 [73]	5	Ø 39,8 (27–53)	60%	UK	July 7, 2005	C	Foreign biological body injuries	6
Yasin 2011 [74]	1,296	Ø 33.4 (± 2.3)	98%	Pakistan	2008–2011	C&M	General injury pattern	10
Yazgan 2016 [75]	28	Mdn 42 (range 18–65)	75%	Turkey	October 10, 2015	C	General Injury pattern	10

*Legend*: ^a^ context: C=civilian, M=military, C&M= mixed population of civilians and military personnel; ^**b**^ Sex: percentage of male victims

JBI= Joanne Briggs Institute; Ø = mean; study ID = study identification; - = not reported; Mdn = median

### General injury pattern

**Fig. 2:**
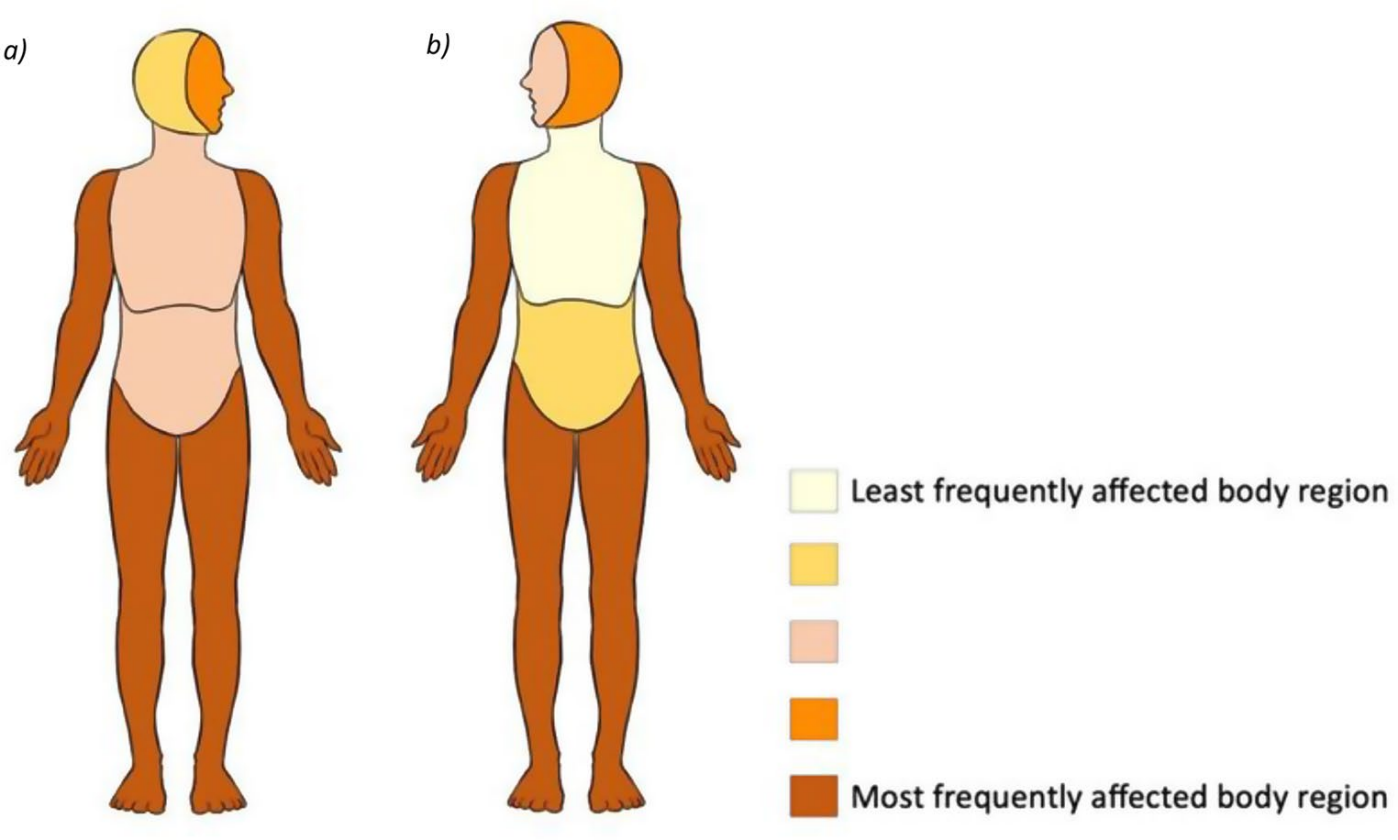
**a**)Civil and military, **b**) Civil population

Figure 2 a and b display the ranking of the frequency of the different affected body regions. The figures are based on the study population of 23 studies reporting injuries to at least four of five AIS body regions (excluding external injuries) with a minimum of six patients [36, 38, 39, 42, 44–47, 55–57, 60–62, 65, 67, 68, 70, 71, 74–77]. As only one study (Schwartz 2009) investigated military personnel, no ranking of affected body regions was performed. In comparison to Fig. 1a and b, Schwartz 2009 showed that the face was most frequently injured [65]. In contrast, the two other populations (civilians only (a) and mixed civilian/military populations (b)) the extremities were injured most frequently. The frequency of facial injuries was higher in civilians than in the mixed civilian/military populations. Nonetheless, head injuries frequently occurred among civilians. 

The heatmap in Table 5 shows that most studies reported facial, thoracic, or abdominal injuries with a high frequency of injuries. A large proportion of injured persons with abdominal injuries was reported by Bala 2008, Franceschetti 2021 and Waterworth 1975. While Bala 2008 reported an analysis of hospital data obtained during different blast events, Franceschetti 2021 focused on forensic data of two specific blast events from Italy and Waterworth 1975 analyzed injured persons of one event in Birmingham in 1974 [1, 42, 76].

**Table 5:**
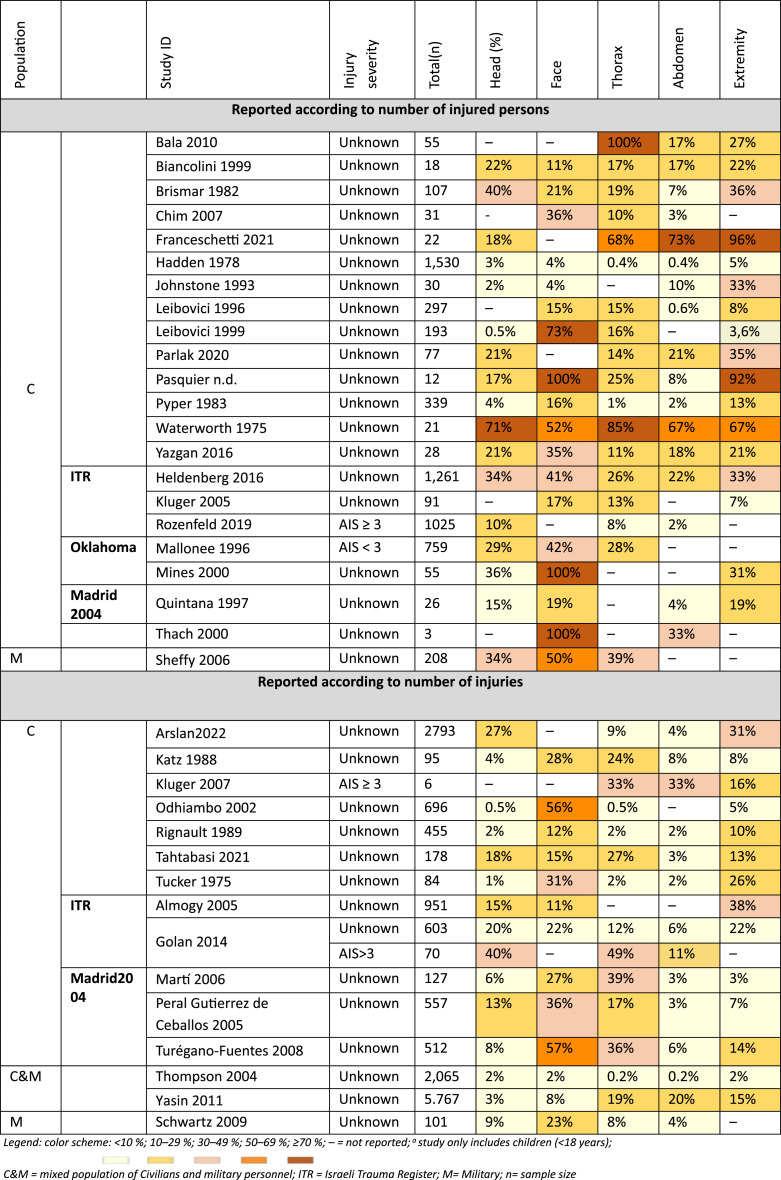
Heatmap terror including suicide bombing

The data indicate that the proportion of head trauma varies widely, from 0.5% as in Leibovici 1999 (diverse terror attacks, Israel, 1994–1996) to 71% in Waterworth 1975 (Birmingham, UK, two public house explosions, 1974), with high rates observed in studies on mass-casualty events, such as 50% (Scott 1986; Beirut Lebanon, Bomb Explosion, 1983) and 40% (Brismar 1982; Bologna, Italy, Bomb Explosion, 1980) [39, 50, 66, 76]. Some studies show very high rates of facial injuries [54, 57, 69] - mostly reported as ear and/or eye injuries, but can also include the jaw and mouth.

Thoracic injuries, on the other hand, seem to appear less frequently, with typically 10–30% of the reported injuries, but can be as high as 85%, as in Waterworth 1975 (Birmingham, UK, two public house explosions, 1974). This study reported a very high rate of injuries to all body parts eventually caused by an explosion in a closed space [76]. Abdominal injuries generally appeared at a low rate (∼10%) with the exception of some studies, such as 73% in Franceschetti 2021 (Milan Italy, Explosion, National Agriculture Bank in December 1969 & Palestro Street 1993) [42].

Extremity injuries are consistently the most frequently injured body region, especially high in Franceschetti 2021 (96%) and Pasquier n.d. (92%), Bala 2008 (86%) [1, 42, 57]. E.g., Pasquier n.d. investigated the injuries of mounted engineers in a bus attacked by a car bomb [57].

Quintana (1997) analyzed children with blast injuries after the Oklahoma bombing in April 1995. The ranking of children is similar to that of adult studies, but soft tissue injuries were the predominant injuries with lacerations, abrasions, fractures, and dislocations (not shown in the heatmap). Nineteen of the 26 children investigated in this study died. Of these casualties, 17 had a massive head injury (skull fractures, 15 of those open, most with complete cerebral evisceration) [61] . More detailed information on the injury patterns within individual studies can be found in Additional File 4.

Lower extremity injuries were shown with a median of 28–30% across the studies, whereas upper extremity injuries occurred less frequently with a median of approximately 18–20%. High rates of upper extremity injuries were shown by Arslan 2022 (Somalia, several suicide bombings, 2014–2021) [36] and Golan 2014 (Israel, series of 22 bombings that involved buses, 2000–2004) [43]. Of the extremity injuries, amputations were reported with a median rate of approximately 12%; however, some studies show rates of up to 30% (Patel 2012). Patel 2012 analyzed specifically traumatic amputations of victims of the London 07/07 bombing [58].

### Focus on specific injuries or body regions

Several studies have focused on body regions or specific injuries in their analyses with more information provided in Additional File 4. For example chest injuries were investigated by Bala 2010 and abdominal injuries by Bala 2008 [1, 37]. Some studies analyzed external injuries e.g., burn injuries in more detail [40, 41, 63]. Also specific facial injuries were studied by some e.g., maxillofacial injuries and eye injuries by Odhiambo 2002, ear injuries by Leibovici 1997 and eye injuries by Mines 2000 and Tach 2000 [50, 54, 55, 69].

## Discussion

### Landmines

For landmines 12 studies reporting injury to victims in many contaminated areas globally were included. These are mainly studies of post-conflict, low- and middle-income countries. The included studies cover the period 1980 to 2006 and represent the years with the highest number of casualties [26], even before mine clearance efforts started [17, 30].

Studies predominantly reported on injuries in civilians, including children [26]. The majority of victims are male, which may be attributed to the fact that, in the location of the included studies, men constitute the economically productive population, earning their living to support their families through agricultural work such as farming, animal grazing, or other outdoor activities [30, 34, 79]. This may also reflect the cultural context of the investigated countries, where women and girls tend to stay indoors more frequently [33]. This is reflected in the average age across all studies (24.0 years) which corresponds with the economically productive age [79] and highlights the high proportion of affected children as also described by Bendinelli and colleagues 2009 for a Cambodian cohort in which one third of affected victims were children [17, 27, 80, 81].

The included studies reported injuries to the extremities in the form of traumatic amputations, and injuries to the eyes [13, 29, 34]. This is in line with the literature that states that landmines inflict severe injuries with the intent of causing morbidity rather than mortality, thereby demoralizing troops while imposing economic and rehabilitation burdens [13].

However, this stands in contrast to the findings of Bendinelli and colleagues 2009, who showed that the injury patterns in children differ tremendously from those in adults. Children mostly suffer from head and torso injuries [80]. This might be caused by children playing with landmines or UXOs and interrelatedly lifting the landmines. They often require major surgery to control injuries to vital organs and have a higher in-hospital mortality rate than adults. The included study by Mousavi 2015, who reported on children exclusively [33] neither confirms nor refutes the injury patterns reported in children by Bendinelli and colleagues 2009 [80]. However, Mousavi 2015 did not report any torso injuries but stated that amputations and hearing loss were the most common injuries in their Iranian cohort, with 80% of injuries caused by landmines [33].

The investigation of injury patterns was complicated by the fact that five studies employed the Coupland classification to describe injury patterns [14, 25, 27, 30]. These studies frequently reported only injuries in relation to the classification, without further details of the injuries. Three of the included studies did not utilize a classification system [13, 32, 34] but attributed the extent and patterns of injuries to the activities performed during detonation. Hence, most details on injury patterns are missing. Nonetheless, face and extremities appear to be the most frequently injured body regions. Most studies on facial injuries focused on eye and ear injuries with e.g., Muzzafar 2000 highlighted the high frequency of blindness due to mine clearing activities [13].

Extremity injuries most frequently include or result in amputations, as reflected by the included studies that focused on specific body parts. Four of five studies report on extremity injuries and more specifically on amputations [14, 26, 28, 31]. Lower extremities appear to be more frequently involved, even though Bilukha 2009 showed a nearly even distribution of lower and upper extremity amputations [26]. This is in line with other studies on blast injuries due to landmines and UXOs [80, 82]. Goonetilleke (1995 and Khan 2002 showed that lower extremity amputations often occur above the ankle [14, 31]. Several of the studies included only reported injuries as the number of injured persons but not the number of amputations. There is a lack of specific details regarding the anatomical location, total number of amputations and the severity of injuries (e.g [14, 31, 33]).

Furthermore, the injury pattern may differ significantly depending on the injury mechanism. Landmines or antipersonnel mines are designed to cause harm and disability to humans. UXOs and (abandoned explosive ordnances (AXOs), on the other hand include a wide range of different explosive munitions such as mines, cluster munitions, unexploded ordnance, abandoned ordnance, booby traps, improvised explosive devices, and other devices which may cause more devastating injuries and mortality than landmines. Morikawa 1998 reported that the mortality rate in their UXO cohort study in Laos did not differ between children and adults. However, males were more likely to die than females. Injuries are often caused by multiple fragments dispersed throughout the body [81]. Moreover, Smith 2017 highlights that the use of conventional landmines has decreased, while the use of (antipersonnel) IEDs has increased. These devices cause far more serious injuries than conventional landmines, as illustrated by the increase in perineal injuries which are rare in landmine victims but common in IED victims [83].

### Terror including suicide bombing

45 studies examining blast injuries from terrorist or suicide attacks were included in this review, with events primarily from the early 2000s. Many incidents have occurred in(post-)conflict regions and cities in high-income countries without ongoing conflict. Hence, most studies have focused on civilian populations, while only a few studies have addressed military personnel and mixed populations. Unlike other trauma mechanisms, victims of terrorist attacks tend to be younger and the gender distribution is nearly equal, as the events typically occur while people are going about their daily lives at places where people congregate e.g., commuting to work, school or running errands [1, 3, 51, 53, 84].

The injured individuals in the included studies exhibited multidimensional injury characteristics resulting from the explosion, including blunt and penetrating trauma, and burns affecting multiple organ systems and body parts [4, 6]. Extremity injuries were the most frequent. This finding is consistent with that of Almogy 2005, who reported soft tissue and bone injuries in nearly 85% of cases [35]. Limb injuries from terrorist events often include fractures, lacerations, and traumatic amputations. Fractures and blunt trauma are primarily caused by tertiary blast effects, which occur when an individual is propelled against a solid object [9]. Additionally, flying, highly accelerated debris (secondary blast effect) can lead to fractures or soft tissue injuries, depending on the size and number of fragments [85, 86]. Depending on their proximity to the explosion, patients may suffer traumatic amputations and other serious, life-threatening injuries [87]. The increased mortality rate due to traumatic amputations reflects the severity of consequences of these explosions [88, 89]. Amputations predominantly occur along the shafts of long bones rather than at the joints [58, 90]. The pathomechanism is explained by the coupling of the blast wave, which induces axial stress that results in those fractures. Subsequently, the blast wind and shrapnel contribute to severe amputations [58, 87, 90, 91]. Therefore, medical responders should prioritize the management of limb trauma, including hemorrhage control, in cases of traumatic amputations. Trauma systems in high-risk regions must be equipped to handle mass casualties characterized by limb-dominant injury patterns, necessitating orthopedic, reconstructive, and rehabilitation support.

The second most commonly injured body regions were the head and the face. The studies reviewed describe injuries to the eyes, ears, and maxillofacial area with varying degrees of detail [55, 69, 71]. Severe eye injuries, such as open globe injuries caused by fragments, such as glass splinters have been reported by Thach 2000 [69]. This is corroborated by the literature on eye injuries resulting from terrorist attacks, which provides detailed descriptions of unusual injury patterns [92]. It is assumed that highly accelerated fragments, resulting from secondary blast effects and composed of materials such as iron or copper, as well as soil contaminants, lead to these serious open eye injuries [92, 93]. Other documented injuries include subconjunctival hemorrhage, lamellar corneal lacerations caused by burns and/or foreign bodies, and eyelid tears [50]. The severity of these injuries is primarily attributed to the absence of protective eyewear [92]. Additionally, complications such as infections and immediate or delayed enucleation must be considered [92, 93]. Ear injuries are often overlooked because of their typically non-visible nature and perceived minor significance compared with other injuries. Some studies report a high incidence of tympanic membrane ruptures following terrorist attacks, affecting the majority of patients (Leibovici 1999, Pasquier, n.d.) [50, 57]. Others have also documented injuries to the middle and inner ear [94–96]. As outlined in a systematic review by Debenham et al., many patients present with additional symptoms such as hearing loss (83%), tinnitus (84.7%), and otalgia (26.6%) [94]. Other studies reported facial injuries including soft tissue injuries, primarily affecting the eyes (20.7%), cheeks (18.1%), and forehead (14.1%), as well as fractures resulting from secondary and tertiary blast effects [55, 71]. This is consistent with previous studies [44].

Thoracic and abdominal injuries, although infrequent in terrorist attacks, are generally associated with higher mortality rates [44, 89]. Despite being partially protected by the rib cage, the thorax can sustain severe injuries due to a combination of blast waves and penetrating shrapnel [37]. Most patients experience lung contusions as a result of pulmonary barotrauma [5]. Patients also experience inhalation trauma [9]. Although the abdomen is less frequently targeted, its involvement often indicates close-range exposure. Bala 2008, included in this systematic review, outlined individual abdominal injuries in terrorist acts, such as alimentary tract injuries involving the stomach, duodenum, and small or large bowel caused by penetrating shrapnel, with injuries to solid organs being relatively uncommon [1, 5].

Unlike landmine explosions, blast injuries associated with terrorist attacks depend heavily on the location of the explosion and the victim’s distance to the detonation site [6, 97, 98]. Those closest to the site may die instantly due to a combination of ballistic and thermal effects, causing fatal injuries such as barotrauma to the lung or intra-abdominal organs [5]. The number of casualties and severity of injuries increases from open spaces to confined areas. For instance, explosions in confined spaces, such as on buses, lead to localized overpressure, which is further amplified by reflection from walls, which prolongs its duration, increases the density of victims and the bombing’s lethality [2, 4, 5, 9].

In contrast, suicide bombings occur more frequently in open spaces and result in a higher rate of penetrating trauma because of the construction of bombs and the location of the detonation [88, 98]. In this review, we did not further differentiate between these details in our analysis, mainly because many studies report several events in one analysis and do not provide enough detail on the single events to further differentiate the analysis. Most injuries appear to be caused by secondary blast effects. Because civilians lack protection, all parts of the body can be affected. This is exacerbated by the additional debris from homemade bombs. However, barotrauma also plays a significant role in explosion-related injuries, in civilian and military settings [99].

## Limitation

### Landmines & UXO

Data on landmines and UXOs/AXOs are often not reliable and not easily available, as the affected countries often suffer from organizational and social disruption [100]. Hence, the reporting is commonly insufficient or due to a different focus, e.g., using the Coupland classification, which is not useful to derive a precise overview of the injury pattern [29]. The data reported in the included studies are predominantly retrospective hospital data, implying that prehospital deaths may not be recorded, potentially leading to an underestimation of the number of injuries [29, 33, 79]. Moreover, due to the high cost of medical treatment, not all casualties, particularly those with minor injuries, may seek medical attention, thereby escaping statistical records [27]. In addition, patients who leave the hospital against medical advice are frequently excluded from the statistics [29].

The mix of populations within the included studies made it difficult to elucidate on differences in injury patterns. Most studies included adults and children [14, 25–30, 32], even though the literature points out that children suffer different injuries than adults, details are often absent [80]. Furthermore, several studies included civilians and military personnel. However these groups possibly suffer from different injuries due to the protective clothing worn by military personnel [14, 25–29].

The search strategy did not capture all landmine articles because the focus was on blast injuries and surrounding synonyms. Hence, a more sophisticated search is needed in future studies to capture all studies, which would then hopefully generate a more complete picture of the injury patterns surrounding landmines and UXOs. According to the literature, this search strategy would need to include terms such as explosive ordnance, mines, cluster munitions, unexploded ordnance, abandoned ordnance, and booby traps.

### Terror attacks and suicide bombings

There is no consistent definition of terrorist attacks and IED attacks, which leads to differential reporting of possibly the same injury mechanism. In the military context, the term IED is frequently used to describe various events, including terrorist attacks. However, the studies included on terror attacks in the present systematic review mainly described civilian populations, with only a few military casualties.

Most studies, particularly those examining terrorism in Israel, relied on retrospective hospital and trauma registry data. These reports frequently overlap in timeframes. Hence, the analyzed population also overlaps. Often, no average injury severity according to the ISS or AIS is reported, which makes comparative analysis and data aggregation difficult.

Furthermore, terrorist attacks frequently result in immediate fatalities at the scene, which are not captured in studies that are solely based on retrospective hospital data, thereby omitting the actual mortality rate, severity of injury and injury patterns that led directly to death. However, knowledge of these types of injuries would help to develop mitigation strategies. Additionally, patients who sustained injuries that were not severe enough to warrant hospital treatment were also excluded from such datasets. This suggests a reporting gap concerning the most and the least severe injuries sustained by those affected by a terror attack or suicide bombing.

## Conclusion

The second part of this systematic review on the patterns of blast injuries focused on landmines including UXOs, and terror attacks, including suicide bombings. The predominant injury region for both explosive mechanisms was the extremities. However, injury patterns varied depending on the injury mechanism, with more facial injuries in landmines, especially in adults, whereas head and thoracic injuries as well as burns, were more common in terror attacks. Therefore, blast injuries are more than just visual wounds. The injury mechanism, distance to the explosive device, and location of detonation determine the pattern and severity of injuries. Furthermore, the characteristics of the victims, such as age and sex, influence the pattern of injuries. Civilians are largely unprotected and hence, also show divergent injury patterns compared to military personnel. Improving the understanding of injury mechanisms and injury patterns will lead to an improved allocation of resources and an increased effectiveness of injury treatment which will ultimately improve the patient care and outcomes.

The high number of civil and, especially, child victims severely injured by landmines highlights the urgent need to raise awareness, improve mine education in the affected countries, and the necessity of mine-clearing operations to decrease the burden of landmines. Furthermore, the results should raise awareness of the patterns and characteristics of blast injuries, especially in non-military and/or post-conflict settings. Training for first responders and medical personnel should emphasize the management of severe limb trauma and hemorrhage control. Preparedness for mass casualties should include sufficient triage, adequate logistics such as tourniquets and blood products, and the provision of medical personnel with expertise in orthopedic trauma, reconstructive surgery, and rehabilitative care.

The reporting of blast injuries is heterogeneous. Following standardized and frequently used recording systems, such as the AIS and the ISS, would improve reporting and leverage the understanding of injuries including their severity. Furthermore, a comprehensive overview, definitions, and terminologies are needed for different explosion mechanisms, especially across populations. This would improve the comparability of the results and, therefore, the usefulness of the knowledge gained from blast research.

## Supplementary Information

Below is the link to the electronic supplementary material.

Supplementary Material 2: Additional file 2: Risk of bias Assessment. shows that risk of bias assessment for all in the entire systematic review included studies.

Supplementary Material 3: Additional file 3: Additional information on landmine & UXO studies. includes tables with additional information on general injury pattern and of injuries to single body regions.

Supplementary Material 4: Additional file 4: PDF. Additional information on terror attacks including suicide bombing. includes tables with additional information on general injury pattern and of injuries to single body regions.

Supplementary Material 1: Additional File 1 - PRISMA checkliste

## Data Availability

All data generated or analyzed during this study are included in this published article and its supplemental information files.

## Abbreviations

AIS: Abbreviated Injury scale
AXO: Abandoned explosive ordnances
C: Civil
C&M: Civil & Military
IED: Improvised explosive devices
ICRC: International Committee of the Red Cross
ISS: Injury Severity Score
ITR: Israel National Trauma Register
JBI: Joanna Briggs Institute
M: Military
Mdn: Median
UXO: Unexploded Ordnance
