# Supplementary material for: Pattern of blast injuries. A systematic review: Part 2 – Landmines, unexploded ordnance and terrorism
Source: Eur J Trauma Emerg Surg. 2026 Jun 18;52(1):201. doi: 10.1007/s00068-026-03241-1 (PMC13279386; doi:10.1007/s00068-026-03241-1)
Supplement: Supplementary file 2 — Supplementary Material 3: Additional file 3: Additional information on landmine & UXO studies. includes tables with additional information on general injury pattern and of injuries to single body regions. [file 68_2026_3241_MOESM2_ESM.docx]

# Additional file 1– additional information landmines & UXO

## General injury pattern

| **Study ID** | **Demographic characteristics** | **Country**  **Time frame** | **Severity of injuries** | **Injury by anatomical region**  **– Nr. of persons with injury (%)** | | **Injuries reported**  Extremity injuries in detail:  **– Nr. of persons with injury (%)** |
| --- | --- | --- | --- | --- | --- | --- |
| **Afshar 2007**  Retrospective hospital data  Children &  adults  Civilian & military | **N=156**  **Age:**  65% 15-35y  **Sex:**  M 148 / F 8  **Civilians:** 125  **Military:** 31  **Multiple injuries:**  -  **Mortality**  **KIA:** -  **DOW:**  6 (4%) | **Iran**  **Time frame:**  1998 - 2004 | Unknown | **Nr. of injured persons:**  **Head**  **Face**  **Thorax**  **Abdomen**  **Extremities**  **Spine**  **Pelvis**  **External**  **Burns** | 156 (100%)  -  35 (22%)  14 (9%)  -  128 (82%)  -  -  22 (14%)  - | **Nr. of injuries:**  238 (100%)  **Upper extremity:**  49 (21%)  **Lower extremity:**  79 (33%)  **Total nr. of amputations:**  110 (46%) |
| **Bilukha 2003**  Retrospective analysis of ICRC data    Children &  Adults  Civilian & military | **N=** 1,636  **Age:**  46% < 16y  **Sex:**  M 1499 /  F 137  **Civilian:** 1329  **Military:** 310  **Multiple injured:**  482 (30%)  **Mortality**  **KIA:** -  **DOW:**  154 (9%) | **Afghanistan**  **Time frame:**  Mach 2001  -  June 2002 | Unknown | **Nr. of injured persons:**  **upper body injuries:**  **lower body injuries:**  **upper and lower body injuries:** | 1,636 (100%)  583 (36%)  391 (24%)  482 (30%) | - |
| **Bilukha 2007**  Information Management System for Mine Action  Children &  adults  Civilian & military | **N= 3,021**  **Age:**  majority  15-24y  **Sex:**  M 2451 /  F 570  **Nr. of multiple injured:**  -  **Mortality:**  **KIA:** -  **DOW:**  687 (23%) | **Chechnya**  **Time frame:**  1994 - 2005 | Unknown | **Nr. of injured persons:**  **Upper body injury:**  **Lower body injury:**  **Upper and lower body injury:** | 3,021 (100%)  840 (28%)  664 (21%)  773 (26%) | **Total nr. of persons with an amputation:**  667 (100%)  **Upper extremity:**  185 (28%)  **Lower extremity:**  482 (72%) |
| **Bilukha 2008**  Retrospective analysis of ICRC data  Children &  adults  Civilian & military | **N= 5,471**  **Age:**  47.2% 0 -17y  **Sex:**  M 4996 /  F 475  **Multiple injuries:**  1,449 (27%)  **Mortality**  **KIA:** -  **DOW:**  939 (17%) | **Afghanistan**  **Time frame:**  January 2002  -  December 2006 | Unknown | **Nr. of injured persons:**  **Upper body injury**  **Lower body injury**  **Upper and lower body injury** | 5,471 (100%)  1,959 (36%)  1,106 (20%)  1,449 (27%) | **Total nr. persons with an amputation:**  2,088 (100%)  **- upper extremity:**  1,055 (51%)  **- lower extremity:**  1,033 (49%) |
| **Coupland 1991**  Retrospective hospital data    Children &  adults  Civilian & military | **N= 757**  **Age: -**  **Sex: -**  **Civilian:** 181  **Military:** 576  **Multiple injured: -**  **Mortality:**  **KIA:** -  **DOW:**  6 (1%) | -  **Time frame:**  1 year | Unknown | **Nr. of injuries:**  **Central injuries (head, neck, chest, abdomen)**  **Face**  **Extremities**  **Spine**  **Pelvis**  **Urogenital** | 1,826 (100%)  415 (23%)  59 (3%)  1,352 (74%)  -  -  49 (3%) | **Nr. of injuries:**  1,352 (100%)  **Upper extremity:**  449 (33%)  **Lower extremity:**  903 (67%)  **Fractures:**  -  **Total nr. of amputations:**  258 (19%) |
| **Damodar 2015**  Retrospective hospital data  Children & adults  Civilian | **N= 54** fatalities  **Age:**  Range 10 – 60  **Sex:**  M 47 / F 7  **Multiple injuries:**  -  **Mortality**  KIA: 50 (93%)  DOW: 4 (7%) | **Vijayawada, India**  **Time frame:** | Unknown | **Number of injuries:**  **Head**  **Face**  **Thorax/Neck**  **Abdomen**  **Extremities**  **Spine**  **Pelvis**  **External** | 142 (100%)  -  2 (1.5%)  11 (8%)  -  76 (54%)  -  -  53 (37%) | **Nr. of injuries:**  76 (100%)  **Upper extremity:**  29 (38%)  **Lower extremity:**  47 (62%) |
| **Goonetilleke 1995**  Retrospective hospital data  Adults  Military | **N= 191**  **Age: -**  **Sex:** M 191 / F 0  **Nr. of multiple injured:** -  **Mortality:**  **KIA:** -  **DOW:** - | **Palaly and Colombo, Sri Lanka**  **Time frame:**  June 1990  -  August 1992 | Unknown | **Numbers of injured persons**  **Head/neck**  **Face**  **Thorax**  **Abdomen**  **Extremities**  **Spine**  **Pelvis**  **External** | 191 (100%)  -  10 (5%)  -  -  153 (80%)  -  -  24 (13%) | **Nr. of persons with an amputation**  153 (100%)  **Upper extremity amputations:**  -  **Lower extremity amputations:**  153 (100%) |
| **Khan 2002**  Prospective cohort study  Children &  adults  Civilian & military | **N= 28**  **Age:**  ∅ 17.75y (13-55)  **Sex:**  M 28 / F 0  **Nr. of multiple injured:** -  **Mortality:**  **KIA:** -  **DOW:** - | **Kashmir Pakistan**  **Time frame:**  - | Unknown | **Number of injured persons:**  **Head/neck**  **Face**  **Thorax**  **Abdomen**  **Extremities**  **Spine**  **Pelvis**  **External**  **Burns** | 28 (100%)  -  -  -  -  28 (100%)  -  -  -  - | **Nr. of injured persons:**  28 (100%)  **With upper extremity:** -  **With lower extremity:**  28 (100%)  **Total nr. of patients with an amputation:**  24 (86%) |
| **Meade 2000**  War Injury Registry  &  Retrospective hospital data  Children &  adults  Civilian | **N= 328**  **Age:**  ∅ 32.2y (6 month - 88)  **Sex:**  M 265 / F 63  **Multiple injuries:**  209 (60%)  **Mortality**  KIA: 45 (12%)  DOW: 11 (3%) | **Jaffna, Sri Lanka**  **Time frame:**  May 1, 1996  -  December 31, 1997 | Unknown | **Number of injuries**  **Head, Neck and Face**  **Thorax and**  **Abdomen**  **Extremities**  **Spine**  **Pelvis**  **External**  **Burns** | 587 (100%)  101 (16%)  104 (18%)  382 (66%)  -  -  -  - | **Nr. of injuries:**  382 (100%)  **Upper extremity:**  134 (35%)  **Lower extremity:**  248 (65%)  **Total nr. of amputations:**  64 (17%) |
| **Mousavi**  **2015**  Iranian  National Veterans Registry  Children  Civilian | **N= 78**  **Age:**  ∅ 8.2y (±3.12)  **Sex:**  M 67 / F 11  **Multiple injured:**  35 (45%)  **Mortality:**  **KIA:** -  **DOW:** - | -  **Time frame:**  1980-1988 | Unknown | **Nr. of injured persons:**  **Head**  **Face**  **Thorax**  **Abdomen**  **Extremities**  **Spine**  **Pelvis**  **External**  **Others** | 78 (100%)  -  45 (58%)  -  -  41 (53%)  3 (7%)  -  -  18 (51%) | **Total nr. of persons with an amputation:**  41 (100%)  **Upper extremity amputation:**  26 (63%)  **Lower extremity amputation:**  17 (38 %)  **Number of persons with at least 2 limb amputations:**  12 (29%) |
| **Muzaffar 2000**  Retrospective hospital data  Adults  Civilians | **N= 51**  **Age:**  ∅ 29y (range 19- 56)  **Sex:**  M 51 / F 0  **Nr. of multiple injured:**  40  **Mortality:**  KIA: -  DOW: - | **Afghanistan**  **Time frame:**  November 1992 to January 1996 | Unknown | **Nr. of injuries:**  **Head/neck**  **Face**  **Thorax**  **Abdomen**  **Extremities**  **Spine**  **Pelvis**  **External** | 91 (100%)  -  91 (100%)  -  -  -  -  -  - | - |
| **Soroush 2008**  retrospective review of hospital data  Children & adults  civilian | **N= 1,499**  **Age:**  ∅ 23y (±13)  **Sex:**  M 1379 /  F 120  **Multiple injured:** -  **Mortality**  KIA: -  DOW: - | **Iran**  **Time frame:**  August 20, 1988  -  March 20, 2003 | Unknown | **Nr. of injuries:**  **Head & neck**  **Face**  **Thorax**  **Abdomen**  **Extremities**  **Spine**  **Pelvis**  **External incl. burns** | 4,153 (100%)  339 (8%)  136 (3%)  61 (1%)  93 (2%)  3,502 (84%)  -  22 (0.5%) | **No. of injuries:**  3,502 (100%)  **Upper extremity:**  597 (17%)  **Lower extremity:** 1,220 (35%)  **Total nr. of amputations:**  1,685 (48%)  **Number of Patients with at least 2 limb amputations:**  285 (19%) |

DOW = died of wounds; F = Female; ICRC = International Committee of the Red Cross; KIA = killed in action; M = male; N = sample size; y= years; - = not reported; ∅ = mean; ± = standard deviation; *Color indicates the same data source: ICRC data*

## Focus on specific injuries

Muzzafar (2000) investigated eye injuries in Afghanistan. They showed that in their collective, patients had predominately both eyes injured (44% of patients with eye injuries) (table below). Of the 91 injured eyes, 34 became totally blind and 42 were left with visual impairment. In comparison to other studies on landmines, this paper concentrated on male non-military adults who are involved in mine clearing operations. None of the patients were wearing protective clothes or eye protection (94).

Four studies focused on amputations (86,87,90,91). As also shown above, amputations are common in patients injured by landmines/UXO. Bilukha and colleagues conducted two analyses of the ICRC register for data on children and adults injured by landmines in Chechnya (86) and in Afghanistan (87). They found that in Chechnya (timeframe: 1994 to 2005) 22% of the total investigated population (n= 3,021) suffered from an amputation, of these the most affected were the lower extremities (n=482, 72% of amputations). Whereas in Afghanistan (timeframe: 2002 – 2005), 38% of the total population (N= 5,471) suffered an amputation. The distribution of affected extremities was nearly even (upper extremities 51%, n=1055; lower extremities 49%, n= 1033).

Further, Goonetilleke 1995 (90), who investigated mostly military landmine injuries in Sri Lanka (timeframe 1990 -1992), used the pattern classification by Coupland (1991) (88) und focused on lower extremity injuries. They showed that 42% of lower extremity injuries affect the foot below the ankle, were as 39% affect the leg above the ankle. Due to the ICRC classification, the study lacks further detail. Khan (2002) investigated hindfoot injuries in landmine victims in Pakistan (n=2) and showed that most amputations were affecting the distal tibia (91).

Table

| **Body part** | **Study ID** | **Severity of injuries** | **Specifics of the Injuries**  **– nr. of injuries (%)** | |
| --- | --- | --- | --- | --- |
| **Eye injuries** | **Muzaffar 2000** | Unknown | **Total eye injuries:**  Patients with one eye damaged:  Patients with both eyes damaged: | **91**  11 (12.1%)  40 (44%) |
| **Lower limb injuries** | **Khan 2002** | Unknown | **Total traumatic lower-limb amputations**  distal tibia amputations:  forefoot amputation: | **26**  19 (73%)  7 (27%) |
| **Amputation** | **Goonetilleke 1995** | Unknown | **Total traumatic lower limb amputations:**  fore foot amputations  distal row of the fore food amputation  amputation trough calcaneum  amputation above the ankle | **153**  6 (3.9%)  34 (22.2%)  24 (15.7%)  59 (38.5%) |
