# Supplementary material for: Pattern of blast injuries. A systematic review: Part 2 – Landmines, unexploded ordnance and terrorism
Source: Eur J Trauma Emerg Surg. 2026 Jun 18;52(1):201. doi: 10.1007/s00068-026-03241-1 (PMC13279386; doi:10.1007/s00068-026-03241-1)
Supplement: Supplementary file 3 — Supplementary Material 4: Additional file 4: PDF. Additional information on terror attacks including suicide bombing. includes tables with additional information on general injury pattern and of injuries to single body regions. [file 68_2026_3241_MOESM3_ESM.docx]

# Additional File 2 – terror & suicide bomber additional information

## A) Overview general injury pattern

| **Study ID** | **Demographic characteristics** | **Country**    **Time frame**    **Cause** | **Severity of injuries** | **Injury by anatomical region** | | **Details of extremity injuries reported** |
| --- | --- | --- | --- | --- | --- | --- |
| **Ahmad 2024**    Retrospective hospital data    Children & Adults    - | **N=** 129    **Age:** range 4-65 (31-40 193 (36.69%))  **Sex:** -    **Multiple injuries:** -    **Mortality:**  **KIA:** -  **DOW:** - | Nigeria    **Time frame:**  January 2009 – December 2021 | **Unknown** | **Nr. of injuries**    **Head:**  **Face:**  **Abdomen:**  **Thorax:**  **Extremity:**  **Spine:**  **Pelvis:**  **External:**  **Urogenital:** | 207 (100%)    -  78 (38%)  -  -  -  -  -  129 (62%)  - | - |
| **Almogy 2005**    Retrospective hospital data & ITR    Adults    Civilians | **N**=951    **Age:** Mdn 38  (range 3 weeks – 85)  **Sex:** -    **Multiple injuries:** -    **Mortality**  **KIA & DOW:** 153 (16%) | Israel    **Time frame:**  April 1994 - August 1997    **Cause:**  Suicide bomber | **Unknown** | **Nr. of injuries**    **Head**  **Face**  **Thorax**  **Abdomen**  **Extremities**  **Spine**  **Pelvis**  **External** | 655 (100%)    95 (15%)  73 (11%)  60 (9%)  -  249 (38%)  -  -  - | **Nr. of injuries**  249 (100%)    **Extremity fractures:**  105 (42%)    **penetrating extremity wounds:**  64 (26%)    **Total nr. of amputations:**  74 (30%) |
| **Arslan2022**    Retrospective hospital data    Adults    Civilian | **N=** 1,073    **Age:** 45-59y 317 (30%)  **Sex:**M 751 / F 322    **Multiple injuries:**  752 (70%)    **Mortality**  **KIA:** -  **DOW:**  132 (16%) | Somalia    **Time frame:**  May 2014 - April 2021    **Cause:**  Terror attack | **Unknown** | **Nr. of injuries**    **Head**  **Face**  **Thorax**  **Abdomen**  **Extremities**  **Spine**  **Pelvis / Trunk**  **External**  **Burns** | **2793**    761 (27%)  -  256 (9%)  118 (4%)  878 (31%)  93 (3%)  182 (7%)  364 (13%)  141 (5%) | **No. of injuries:**  878 (100%)    **Upper extremity:**  403 (38%)    **Lower extremity:**  475 (54%) |
| **Bala 2008**    Retrospective hospital data    Adults    Civilians | **N=** 21    **Age:** Mdn 21  (IQR 17-28)  **Sex:** M 12 /F 9    **Multiple injuries:**  18 (86%)    **Mortality:**  **KIA**: -  **DOW:**  4 (19%) | Israel    **Time frame:**  October 2000 -December 2005    **Cause:**  Terrorist bombing attack | **Unknown** | **Nr. of persons injured:**    **Head/neck**  **Face**  **Thorax**  **Abdomen**  **Extremities**  **Spine**  **Pelvis**  **External**  **Burns** | 21 (100%)    -  3 (14%)  -  18 (86%)  -  -  -  -  6 (29%) | - |
| **Bala 2010**    Retrospective hospital data    Adults    Civilian | **N**=55    **Age**: Mdn 28  (IQR 20-35)  **Sex:**  M 28 /F 27    **Multiple injuries:**  43 (78%)    **Mortality**  **KIA:** -  **DOW:**  6 (11%) | Israel    **Time frame:**  October 2000 - December 2005    **Cause:**  Terrorist bombing attack | **Unknown** | **Nr. of persons injured:**    **Head**  **Face**  **Thorax**  **Abdomen**  **Extremities**  **Spine**  **Pelvis**  **External**  **Burns** | 55 (100%)    -  -  55 (100%)  9 (17%)  15 (27%)  1 (2%)  -  -  - | **No. of injuries:**  15    **Lower extremity:**  2 (13%)    **Fractures:**  2 (13%) |
| **Biancolini 1999**   Retrospective hospital data    Adults & children    Civilians | **N**= 18    **Age:**  Ø 34 (±18)  **Sex:** -    **Multiple injuries:**  17 (20%)    **Mortality**  **KIA:** 2 (2%)  **DOW:** 7 (8%) | Buenos Aires, Argentina    **Time frame:**  July 18, 1994    **Cause:**  Car bomb | **Unknown** | **Nr. of persons injured:**    **Head**  **Face**  **Thorax**  **Abdomen**  **Extremities**  **Spine**  **Pelvis**  **External**  **Burns** | 18 (100%)    4 (22%)  2 (11%)  3 (17%)  3 (17%)  4 (22%)  -  -  -  1 (6%) | **Nr. of injuries**  4    **Upper extremity injuries:**  2 (50%)    **Lower extremity injuries:**  2 (50%)    **Total nr. of amputations:**  2 (50%) |
| **Brismar 1982**    Retrospective hospital data    Adults & children    Civilian | **N**= 107    **Age:** Ø 27  **Sex:** M 66 /F 41    **Multiple injuries:** -    **Mortality**  **KIA:** -  **DOW**: - | Bologna, Italy    **Time frame:**  August 02, 1980    **Cause:**  Suite case bomb | **Unknown** | **Nr. of persons injured:**    **Head**  **Face**  **Thorax**  **Abdomen**  **Extremities**  **Spine**  **Pelvis**  **External**  **Burns** | 107 (100%)    43 (40%)  22 (21%)  20 (19%)  7 (7%)  38 (36%)  7 (7%)  -  72 (67%)  28 (26%) | **Nr. of persons with injuries**  38 (100%)    **Upper extremity injuries:**  20 (53%)    **Lower extremity injuries:**  18 (47%)    **Total nr. of amputations:**  3 (8%) |
| **Chim 2007**    Retrospective hospital data    Adults & children    Civilians | **N=** 31    **Age:** Mdn 32 (range 13-56)  **Sex:** M 17 /F 14    **Multiple injuries:** -    **Mortality**  **KIA:** -  **DOW:** 1 (3%) | Southeast Asia    **Time frame:**  October 2002, August 2005 & October 2005    **Cause:**  Suicide bomb | **Unknown** | **Nr. of injured persons:**    **Head**  **Face**  **Thorax**  **Abdomen**  **Extremities**  **Spine**  **Pelvis**  **External**  **Burns** | 31 (100%)    -  11 (36%)  3 (10%)  1 (3%)  -  -  -  7 (23%)  31 (100%) |  |
| **Chukwu-Lobelu 2017**    Retrospective review of casualty data    -    Civilians | **N**= 52    **Age:** -  **Sex:** -    **Multiple injuries:** -    **Mortality:**  **KIA:** -  **DOW:** - | London, UK    **Time frame:**  July 7, 2005    **Cause:**  Suicide bomb | **Unknown** | **Nr. of injured persons**    **Head/neck**  **Face**  **Thorax**  **Abdomen**  **Extremities**  **Spine**  **Pelvis**  **External**  **Burns** | 52 (100%)    -  -  -  -  -  -  -  -  52 (100%) | - |
| **Franceschetti 2021**    Retrospective hospital data    -    Civilians | **N=** 22    **Age:** -  **Sex:** -    **Multiple injuries:**  22 (100%)    **Mortality:**  Paper reports only on fatalities  **KIA:** -  **DOW:** 22 (100%) | Milan, Italy    **Time frame:**  December 1969 &   July 1993    **Cause:**  Terrorist bombing attack | **Unknown** | **Nr. of injured persons:**    **Head/neck**  **Face**  **Thorax**  **Abdomen**  **Extremities**  **Spine**  **Pelvis**  **External**  **Burns** | 22 (100%)    4 (18%)  -  15 (68%)  16 (73%)  21 (96%)  -  -  21 (96%)  17 (77%) | **Nr. of extremity injuries**  38 (100%)    **Upper extremity injuries:**  10 (26%)    **Lower extremity injuries:**  5 (13%)    **Total nr. of amputations:**  26 (68%) |
| **Golan 2014**    ITR    Adults    Civilian | **N=** 262    **Age:** -  **Sex:** -    **Multiple injuries:**  106 (40%)    **Mortality**  **KIA:** -  **DOW:** 15 (6%) | Israel    **Time frame:**  November 2000 & August 2004    **Cause:**  Terror attack | **AIS > 3** | **Nr. of body regions injured:**    **Head & neck**  **Face**  **Thorax**  **Abdomen**  **Extremities**  **Spine**  **Pelvis**  **External**  **Burns** | 70 (100%)    28 (40%)  0 (0%)  34 (48.5%)  8 (11.4%)  0 (0%)  -  -  0 (0%)  - | **No. of injuries:**  135 (100%)    **Upper extremity:**  92 (68%)    **Lower extremity:** 83 (62%) |
|  |  |  | **Unknown** | **Nr. of body regions injured:**    **Head & neck**  **Face**  **Thorax**  **Abdomen**  **Extremities**  **Spine**  **Pelvis**  **External**  **Burns** | 603 (100%)    117 (20%)  134 (22%)  72 (12%)  38 (6%)  135 (22.4%)  -  -  107 (18%)  - |  |
| **Hadden 1978**    Retrospective hospital data    Civilians    Adults & children | **N=** 1,532    **Age:** -  **Sex:** M 613/ F 919    **Multiple injuries:** -    **Mortality**  **KIA:** -  **DOW:**  9 (0.5%) | North Ireland    **Time frame:**  1969 - 1972    **Cause:**  Terrorist bombing and booby traps | **Unknown** | **Nr. of injured persons:**    **Head**  **Face**  **Thorax**  **Abdomen**  **Extremities**  **Spine**  **Pelvis**  **External**  **Burns** | 1,532 (100%)    39 (3%)  55 (4%)  5 (0.4%)  6 (0.4%)  70 (5%)  1 (0.1%)  -  1098 (72%)  50 (3%) | **Nr. of injured persons:**  70    **Upper extremity injuries:**  18 (26%)    **Lower extremity injuries:**  32 (46%)    **Total nr. of amputations:**  20 (29%) |
| **Heldenberg 2016**    ITR    Adults & children    Civilians | **N=** 1,261    **Age:** majority between 15-29  **Sex:** M 870 /  F 391    **Multiple injuries:** -    **Mortality:**  **KIA:** -  **DOW:** - | Israel    **Time frame:**  September 2000 - December 2005    **Cause:**  Terrorist attacks | **Unknown** | **Nr. of persons injured:**    **Head/neck**  **Face**  **Thorax**  **Abdomen**  **Upper Extremities**  **Lower Extremities**  **Spine**  **Pelvis**  **External**  **Burns** | 1,261    432 (34%)  515 (41%)  331 (26%)  283 (22%)  416 (33%)  481 (38%)  -  -  373 (30%)  - | **Nr. of persons with extremity injuries**  897 (100%)    **Upper extremity injuries:**  416 (46%)    **Lower extremity injuries:**  481 (38%) |
| **Johnstone 1993**    Retrospective hospital data    Adults & children    Civilians | **N=** 30    **Age:** Ø 29.1 (range 12-56)  **Sex:** M 14/ F 26    **Multiple injuries:** -    **Mortality**  **KIA:** -  **DOW:** 1 (2%) | London, UK    **Time frame:**  February 18, 1991    **Cause:**  Litter bin bomb | **Unknown** | **Nr. of persons injured:**    **Head**  **Face**  **Thorax**  **Abdomen**  **Extremities**  **Spine**  **Pelvis**  **External**  **Burns** | 30 (100%)    1 (2%)  2 (4%)  -  5 (10%)  10 (33%)  -  -  32 (64%)  0 | **Nr. of persons with extremity injuries:**  10 (100%)    **Upper extremity injuries:**  2 (20%)    **Lower extremity injuries:**  8 (80%)    **Total nr. of amputations:**  2 (20%) |
| **Katz 1988**    Retrospective hospital data    Civilians    Adults & children | **N**= 29    **Age:** -  **Sex:** -    **Multiple injuries:**  8 (10%)    **Mortality**  **KIA**: -  **DOW**: 3 (10%) | Israel    **Time frame:**  -    **Cause:**  Bus bomb | **Unknown** | **Nr. of body regions injured:**    **Head**  **Face**  **Thorax**  **Abdomen**  **Extremities**  **Spine**  **Pelvis**  **External**  **Burns** | 29 (100%)    4 (14%)  27 (93%)  23 (79%)  4 (14%)  8 (28%)  -  -  20 (69%)  5 (17%) | **Nr. of persons with injuries**  8 (100%)    **Limb fractures:**  7 (88%)    **Total nr. of amputations:**  1 (13%) |
| **Kluger 2005**    Retrospective  Medical Corps of the Israeli Defense Forces; ITR, National Centre of Forensic Medicine    Adults and children    Civilian | **N**= 91    **Age:** Ø 65 (±27,3)  **Sex:** -    **Multiple injuries:**  71 (78%)    **Mortality**  **KIA:** 20 (22%)  **DOW:**  10 (11%) | Israel    **Time frame:**  2002    **Cause:**  Suicide bombing with 8-10 kg charge and metal balls | **Unknown** | **Nr. of persons injured (survivors):**    **Head**  **Face**  **Thorax**  **Abdomen**  **Extremities**  **Spine**  **Pelvis**  **External**  **Burns** | 71 (100%)    -  12 (17%)  9 (13%)  -  5 (7%)  -  -  -  12 (17%) | **Nr. of persons injured:**  5 (100%)  **Upper extremity injuries:**  4 (80%)    **Lower extremity injuries:**  - |
| **Kluger 2007**    Case report    Adults    Civilian | **N=** 4    **Age:** Ø 30.2  (range 24-38)  **Sex:** M 4 /F 0    **Multiple injured:**  3 (11%)    **Mortality:**  **KIA:** 0 (0%)  **DOW:** 3 (11%) | Israel    **Time frame:**  April 30, 2003    **Cause:**  Suicide bomb | **AIS < 3** | **Nr. of injuries:**    **Head**  **Face**  **Thorax**  **Abdomen**  **Extremities**  **Spine**  **Pelvis**  **External**  **Burns** | 7 (100%)    1 (14%)  6 (85%)  0 (0%)  0 (0%)  0 (0%)  0 (0%)  0 (0%)  0 (0%)  0 (0%) | **Nr. of injuries**  1 (100%)      **Fractures:**  1 (100%)    **Total nr. of amputations:**  1 (100%) |
|  |  |  | **AIS ≥ 3** | **Nr. of injuries**    **Head**  **Face**  **Thorax**  **Abdomen**  **Extremities**  **Spine**  **Pelvis**  **External**  **Burns** | 6 (100%)    0 (0%)  0 (0%)  2 (33%)  2 (33%)  1 (16%)  0 (0%)  0 (0%)  0 (0%)  1 (16%) |  |
| **Leibovici 1996**    Retrospective hospital data    Adults & children    Civilians | **N=** 297    **Age:**  range 3-82  **Sex:**  M 161 / F 135    **Multiple injuries:** -    **Mortality**  **KIA:** -  **DOW:**  61 (21%) | Israel    **Time frame:**  1996    **Cause:**  terrorist bombing | **Unknown** | **Nr. of injured person:**    **Head**  **Face**  **Thorax**  **Abdomen**  **Extremities**  **Spine**  **Pelvis**  **External**  **Burns** | 297 (100%)    -  44 (15%)  43 (15%)  2 (0.6%)  24 (8%)  -  -  29 (10%)  68 (23%) | **Nr. of injuries**  24 (100%)      **Total nr. of amputations:**  24 (100%) |
| **Leibovici 1999**    Retrospective hospital data    Adults & children    Civilians | **N=** 193    **Age:** Ø 31.8 (range 8-81)  **Sex:**  M 98 /F 95    **Multiple injuries:**-    **Mortality**  **KIA:** -  **DOW:** 8 (4%) | Israel      **Time frame:**  April 1994 – March 1996    **Cause:**  Suicide bomb | **Unknown** | **Nr. of injured persons:**    **Head**  **Face**  **Thorax/Abdomen**  **Extremities**  **Spine**  **Pelvis**  **External**  **Burns** | 193 (100%)    1 (0.5%)  142 (73%)  31 (16%)  7 (3.6%)  -  -  -  25 (12.8%) | **Nr. of persons with injuries**  7 (100%)    **Limb fractures:**  5 (71%)    **Total nr. of amputations:**  2 (29%) |
| **Mallonee 1996**    Retrospective hospital data    Civilians    Adults & children | **N=** 759    **Age:**  Mdn 39 (84% 20-64)  **Sex:**  M 326 /F 433    **Multiple injuries:**  144 (19%)    **Mortality**  **KIA:**  162 (21%)  **DOW:**   5 (0.6%) | Oklahoma City, USA      **Time frame:**  April 19, 1995    **Cause:**  Terrorist bombing | **Unknown** | **Nr. of injured persons:**    **Head**  **Face**  **Thorax**  **Abdomen**  **Upper Extremities**  **Lower Extremities**  **Spine**  **Pelvis**  **External**  **Burns** | 759 (100%)    58 (8%)  191 (25%)  13 (2%)  4 (0.5%)  23 (3%)  24 (3%)  1 (0.1%)  -  506 (67%)  9 (1%) | **Nr. of persons with extremity**  **injuries**  47 (100%)    **Upper extremity injuries:**  23 (49%)    **Lower extremity injuries:**  24 (51%) |
|  |  |  | **AIS > 3** | **Nr. of injured persons:**    **Head**  **Face**  **Thorax**  **Abdomen**  **Extremities**  **Spine**  **Pelvis**  **External**  **Burns** | 759 (100%)    8 (1%)  -  -  -  -  -  -  -  - |  |
| **Martí 2006**    Retrospective hospital data    Adults    Civilian | **N=** 36    **Age:** Ø 37  (range 16-57)  **Sex:** M 20 /F 16    **Multiple injuries:** -    **Mortality**  **KIA:** -  **DOW**: 1 (3%) | Madrid, Spain    **Time frame:**  March 11, 2004    **Cause:**  Train bombing | **Unknown** | **Nr. of injuries:**    **Head**  **Face**  **Thorax**  **Abdomen**  **Extremities**  **Spine**  **Pelvis**  **External**  **Burns** | 127 (100%)    7 (6%)  34 (27%)  49 (39%)  4 (3%)  4 (3%)  2 (2%)  -  23 (18%)  4 (3%) | - |
| **Mines 2000**  Case series  Adults & children    Civilians | **N=** 55    **Age:** Ø 35 (range 1-65)  **Sex:**  M 27 /F 28    **Multiple injuries:**  55 (100%)    **Mortality**  **KIA:** -  **DOW:** - | Oklahoma City, USA      **Time frame:**  April 19, 1995    **Cause:**  Terrorist bombing | **Unknown** | **Nr. of injured persons:**    **Head**  **Face**  **Thorax**  **Abdomen**  **Extremities**  **Spine**  **Pelvis**  **External**  **Burns** | 55 (100%)    20 (36%)  55 (100%)  -  -  17 (31%)  1 (1.8%)  -  -  - | **Nr. of persons with injuries**  17 (100%)    **Upper extremity injuries:**  10 (59%)    **Lower extremity injuries:**  7 (41%) |
| **Odhiambo 2002**    Retrospective hospital data    Adults    Civilian | **N=** 290    **Age:** Ø 31.6 (18-66)  **Sex:**  M 188 /F 102    **Multiple injuries:** -    **Mortality**  **KIA:** -  **DOW:** - | Nairobi, Kenia    **Time frame:**  August 7, 1998    **Cause:**  Car bomb | **Unknown** | **Nr. of injuries:**    **Head**  **Face**  **Thorax**  **Abdomen**  **Extremities**  **Spine**  **Pelvis**  **External**  **Burns** | 696 (100%)    3 (0.5%)  386 (56%)  3 (0.5%)  -  31 (5%)  2 (0.5%)  -  271 (39%)  - | **Nr. of injuries**  31 (100%)    **Upper extremity injuries:**  5 (16%)    **Lower extremity injuries:**  26 (84%) |
| **Parlak 2020**    Retrospective hospital data    Adults and children    Civilians | **N=** 77    **Age:** Ø 34.6  (range 15-75)  **Sex:**  M 57 /F 20    **Multiple injuries:** -    **Mortality:**  **KIA:** -  **DOW:** - | Ankara, Turkey    **Time frame:**  October 10, 2015    **Cause:**  Suicide bomb | **Unknown** | **Nr. of injured persons:**    **Head/neck**  **Face**  **Thorax**  **Abdomen**  **Extremities**  **Spine/pelvis**  **External**  **Burns** | 77 (100%)    8 (10%)  18 (23%)  10 (13%)  19 (25%)  34 (44%)  1 (1.3%)  -  3 (4%) | **Nr. of injured persons:**  34 (100%)    **Upper extremity injuries:**  5 (15%)    **Lower extremity injuries:**  27 (80%)    **Total nr. of amputations:**  2 (7%) |
| **Pasquier n.d.**  Retrospective survey    Adults    Civilians | **N**= 12    **Age: -**  **Sex:** -    **Multiple injuries:** 11 (92%)    **Mortality**  Only survivors reported  **KIA:** -  **DOW:** - | Pakistan    **Time frame:**  May 08, 2002  **Cause:**  Car bomb | **Unknown** | **Nr. of injured persons:**    **Head**  **Face**  **Thorax**  **Abdomen**  **Extremities**  **Spine**  **Pelvis**  **External**  **Burns** | 12 (100%)    2 (17%)  12 (100%)  3 (25%)  1 (8%)  11 (92%)  2 (17%)  -  10 (83%)  - | **Nr. of injuries:**  24 (100%)    **Upper extremity injuries:**  4 (16%)    **Lower extremity injuries:**  20 (83%) |
| **Patel 2012**    Retrospective hospital data    -    Civilians | **N**= 222    **Age:** -  **Sex:** -    **Multiple injuries:** -    **Mortality:**  56 (25%) | London    **Time frame:**  July 7,  2005    **Cause:**  Suicide bombing in bus and underground | **Unknown** | **Nr. of injured persons:**    **Head/neck**  **Face**  **Thorax**  **Abdomen**  **Extremities**  **Spine**  **Pelvis**  **External**  **Burns** | 222 (100%)    -  -  -  -  69 (31%)  -  -  -  - | **Nr. of injuries**  90 (100%)    **Upper extremity amputations:**  19 (21%)    **Lower extremity amputations:**  71 (79%)    **Total nr. of amputations:**  90 (100%) |
| **Peral-Gutierrez de Ceballos 2005**    Retrospective hospital data    Adults and children    Civilians | **N=** 243    **Age:** Ø 32  (range 14-63)  **Sex:**  M 143 / F 100    **Multiple injuries:** -    **Mortality:**  **KIA:** -  **DOW**: 5 (2%) | Madrid, Spain    **Time frame:**  March 11, 2004    **Cause:**  Train bombing | **Unknown** | **Nr. of injuries:**    **Head**  **Face**  **Thorax**  **Abdomen**  **Extremities**  **Spine**  **Pelvis**  **External**  **Burns** | 557 (100%)    70 (13%)  198 (36%)  97 (17%)  15 (3%)  38 (7%)  5 (1%)  -  89 (16%)  45 (8%) | **Nr. of extremity injuries:**  28 (100%)      **Total nr. of amputations:**  2 (7%) |
| **Pyper 1983**    Retrospective hospital data    Civilians    Adults & children | **N=** 339    **Age:** -  **Sex:** -    **Multiple injuries:** -    **Mortality:**  **KIA**: -  **DOW:** - | Northern Ireland    **Time frame:**  1972 - 1980    **Cause:**  Terrorist bombing | **Unknown** | **Nr. of injured person:**    **Head**  **Face**  **Thorax**  **Abdomen**  **Extremities**  **Spine**  **Pelvis**  **External**  **Burns** | 339 (100%)    13 (4%)  53 (16%)  4 (1%)  8 (2%)  45 (13%)  1 (0.2%)  3 (0.8%)  253 (75%)  6 (2%) | **Nr. of extremity injuries:**  100 (100%)    **Upper extremity injuries:**  34 (34%)    **Lower extremity injuries:**  66 (66%)    **Total nr. of amputations:**  13 (13%) |
| **Quintana 1997**    Retrospective hospital data    Children    Civilian | **N=** 26    **Age:**  3 months –15 y  **Sex:** M 17 /F 9    **Multiple injured:**7 (27%)    **Mortality:**  **KIA:** 19 (%)  **DOW:** 0 (0%) | Oklahoma City, USA    **Time frame:**  April 19, 1995    **Cause:**  Truck bomb | **Unknown** | **Nr. of injured persons:**    **Head**  **Face**  **Thorax**  **Abdomen**  **Extremities**  **Spine**  **Pelvis**  **External**  **Burns** | 26 (100%)    4 (15%)  5 (19%)  -  1 (4%)  5 (19%)  -  -  18 (69%)  4 (15%) | **Nr. of extremity injuries:**  6 (100%)    **Upper extremity injuries:**  6 (100%)    **Lower extremity injuries:**  0 (0%) |
| **Rignault 1989**    Retrospective hospital data    Civilians    Adults & children | **N=** 205    **Age:**  Ø 34.5 (range few month – 89y)  **Sex:**M 99 /F 106    **Multiple injuries:**  96 (47%)    **Mortality**  **KIA:** -  **DOW:** 7 (3%) | Paris, France    **Time frame:**  1985 - 1986    **Cause:**  Terrorist bombing | **Unknown** | **Nr. of injuries:**    **Head**  **Face**  **Thorax**  **Abdomen**  **Extremities**  **Spine**  **Pelvis**  **External**  **Burns** | 455 (100%)    11 (2%)  53 (12%)  9 (2%)  8 (2%)  47 (10%)  -  -  294 (65%)  33 (7%) | **Nr. of extremity injuries:**  47 (100%)    **Upper extremity injuries:**  18 (38%)    **Lower extremity injuries:**  23 (48%)    **Total nr. of amputations:**  6 (13%) |
| **Rosenberg 1982**  Retrospective hospital data    Civilians    Adults & children | **N**= 12    **Age:**  range 6-71  **Sex:** -    **Multiple injuries:** -    **Mortality**  **KIA:** -  **DOW:** 1 (8%) | Jerusalem, Israel    **Time frame:**  1975 - 1976    **Cause:**  Terrorist bombing | **Unknown** | **Nr. of injured persons:**    **Head**  **Face**  **Thorax**  **Abdomen**  **Extremities**  **Spine**  **Pelvis**  **External**  **Burns** | 12 (100%)    -  7 (58%)  -  -  1 (8%)  -  -  5 (42%)  12 (100%) | **Nr. of extremity injuries:**  1 (100%)    **Upper extremity injuries:** -    **Lower extremity injuries:**  1 (100%) |
| Rozenfeld 2019 | **N=** 1,025    **Age:**  range 0-60 (43% (437 15-29 y)  **Sex:** M 614 /F 411    **Multiple injuries:** 351 (34%) ≥ 3 injured body regions    **Mortality:**  **KIA:** -  **DOW:** 73 (7%) | Israel    **Time frame:**  January 1997  - December 2016    **Cause:**  Terrorist attacks | **AIS ≥ 3** | **Total nr. of injured persons (all AIS):**    **Head/neck**  **Face**  **Thorax**  **Abdomen**  **Extremities**  **Spine**  **Pelvis**  **External**  **Burns** | 1025 (100%)    104 (10%)  -  85 (8%)  49 (2%)  -  -  -  -  - | **Nr. of persons with extremity injuries:**  740 (100%)    **Nr. of persons with upper extremity injuries:**  332 (45%)    **Nr. of persons with lower extremity injuries:**  408 (40%) |
|  |  |  | **AIS < 3** | **Total nr. of injured persons (all AIS):**    **Head/neck**  **Face**  **Thorax**  **Abdomen**  **Upper Extremities**  **Lower Extremity**  **Spine**  **Pelvis**  **External**  **Burns**  **Others** | 1025 (100%)      295 (29%)  428 (42%)  282 (28%)  241 (24%)  332 (32%)  408 (40%)  45  -  -  -  83 (8%) vascular injuries |  |
| **Schwartz 2009**    Retrospective hospital data    Adults    Military | **N=** 76    **Age:** -  **Sex:** -    **Multiple injuries:** -    **Mortality:**  **KIA**: -  **DOW:** - | Zikim base.  Israel    **Time frame:**  September 11, 2007    **Cause:**  "Kassam" rocket | **Unknown** | **Nr. of injuries:**    **Head/neck**  **Face**  **Thorax**  **Abdomen**  **Upper Extremities**  **Lower extremities**  **Spine**  **Pelvis**  **External**  **Burns** | 101 (100%)    9 (9%)  23 (23%)  8 (8%)  4 (4%)  25 (25%)  23 (23%)  9 (9%)  -  -  - | **Nr. of extremity injuries:**  48 (100%)    **Upper extremity injuries:**  23 (48%)    **Lower extremity injuries:**  25 (52%) |
| **Scott 1986**  Retrospective hospital data    Adults    Military | **N=** 346    **Age:** -  **Sex:** -    **Multiple injuries:** -    **Mortality**  **KIA:** 234 (68%)  **DOW:** 7 (2%) | Beirut,  Lebanon    **Time frame:**  October 23, 1983    **Cause:**  Terrorist bombing | **Unknown** | **Nr. of injured persons:**    **Head**  **Face**  **Thorax**  **Abdomen**  **Extremities**  **Spine**  **Pelvis**  **External**  **Burns**  **peripheral nerve injuries** | 346 (100%)    174 (50%)  31 (9%)  -  -  -  24 (7%)  -  -  -    9 (3%) | - |
| **Sheffy 2006**    Retrospective hospital data    Adults    Civilian & military | **N**= 208    **Age:** Ø 28 (±14.6)  **Sex:**  M 125 /F83    **Multiple injuries:**  83 (40%)    **Mortality:**  **KIA:** -  **DOW:** 12 (6%) | Israel    **Time frame:**  September 29, 2000  -  December 31, 2004    **Cause:**  Terror related missiles | **Unknown** | **Nr. of injured persons:**    **Head/neck**  **Face**  **Thorax**  **Abdomen**  **Upper Extremities**  **Lower extremities**  **Spine**  **Pelvis**  **External**  **Burns** | 208 (100%)    70 (34%)  103 (50%)  80 (39%)  47 (23%)  87 (42%)  99 (48%)  18 (9%)  -  -  54 (26%) | **Nr. of extremity injuries**  186 (100%)    **Upper extremity injuries:**  87 (47%)    **Lower extremity injuries:**  99 (53%) |
| **Tahtabasi 2021a**    Retrospective hospital data    Adults and children    Civilians | **N=** 63    **Age:** Ø 28.6  (± 10.2)  **Sex:**  M 39 /F 24    **Multiple injuries:** -    **Mortality:**  **KIA:** -  **DOW:** - | Mogadishu, Somalia    **Time frame:**  December 28, 2019    **Cause:**  Car bomb | **Unknown** | **Nr. of injuries:**    **Head/neck**  **Face**  **Thorax**  **Abdomen**  **Extremities**  **Spine**  **Pelvis/sacral**  **External**  **Burns**  **Urogenital**  **Vascular injuries** | 178 (100%)    32 (18%)  26 (15%)  48 (27%)  6 (3%)  23 (13%)  2 (1%)  2 (1%)  34(19%)  2 (1%)  1 (0.5%)  2 (1%) | **Nr. of persons with extremity injuries**  23 (100%)    **Upper extremity injuries:**  9 (39%)    **Lower extremity injuries:**  14 (61%) |
| **Thach 2000**  Case series  Military    Adults | **N=** 3    **Age:**  Ø 33 (22-40)  **Sex:** M 2 / F 1    **Multiple injuries:** -    **Mortality**  **KIA:** -  **DOW:** - | Dharan  Saudi Arabia  **Time frame:**  June 25, 1996    **Cause:**  Terrorist bombing | **Unknown** | **Nr. of injured persons:**    **Head**  **Face**  **Thorax**  **Abdomen**  **Extremities**  **Spine**  **Pelvis**  **External**  **Burns** | 3 (100%)    0 (0%)  3 (100%)  0 (0%)  0 (0%)  1 (33%)  0 (0%)  0 (0%)  0 (0%)  0 (0%) | **Nr. of injuries**  1 (100%)    **Upper extremity injuries:**  1 (100%)    **Lower extremity injuries:**  0 (0%) |
| **Thompson**  **2004**    Survey & retrospective hospital data    Adults    Civilian & military | **N=** 420    **Age:**  Ø 32 (± 5.9y)  **Sex:** -    **Multiple injuries:**17 (4%)    **Mortality:**  **KIA:** 19 (5%)  **DOW:** - | Dhahran, Saudi Arabia    **Time frame:**  June 25, 1996    **Cause:**  Truck bomb | **Unknown** | **Nr. of injuries**    **Head/neck**  **Face**  **Thorax**  **Abdomen/Pelvis**  **Extremities**  **Spine**  **External**  **Burns** | 2065 (100%)    40 (2%)  42 (2%)  4 (0.2%)  5 (0.2%)  50 (2%)  27 (1%)  1860 (90%)  - | **Nr. of extremity injuries:**  50 (100%)    **Upper extremity injuries:**  28 (56%)    **Lower extremity injuries:**  22 (44%) |
| **Tucker 1975**  Retrospective hospital data    Adults & children    Civilian | **N=** 37    **Age:** -  **Sex:** -    **Multiple injuries:**10 (53%)    **Mortality**  **KIA:** -  **DOW:** 1 (3%) | London,  UK    **Time frame:**  1975    **Cause:**  Terrorist bombing | **Unknown** | **Nr. of injuries:**    **Head**  **Face**  **Thorax**  **Abdomen**  **Extremities**  **Spine**  **Pelvis**  **External**  **Burns** | 84 (100%)    1 (1%)  26 (31%)  2 (2%)  2 (2%)  22 (26%)  -  1 (1%)  20 (23%)  10 (12%) | **Nr. of extremity injuries:**  22 (100%)    **Upper extremity injuries:**  1 (5%)    **Lower extremity injuries:**  2 (9%)    **Total nr. of amputations:**  2 (9%) |
| **Turégano-Fuentes 2008**    Retrospective hospital data    -    Civilians | **N=** 512    **Age:** -  **Sex:** -    **Multiple injuries:** -  **Mortality:**  **191**  **KIA:** 177 of 2000 total casualties (9%)  **DOW:** 14 (3%) | Madrid, Spain    **Time frame:**  March 11, 2004    **Cause:**  Train bomb | **Unknown** | **Nr. of injured persons:**    **Head/neck**  **Face**  **Thorax**  **Abdomen**  **Extremities**  **Spine**  **Pelvis**  **External**  **Burns** | 512 (100%)    43 (8%)  291 (57%)  184 (36%)  28 (6%)  71 (14%)  25 (5%)  1 (0.2%)  211 (41%)  89 (17%) | **Nr. of extremity injuries**  71 (100%)    **Upper extremity injuries:**  22 (29%)    **Lower extremity injuries:**  38 (54%)    **Total nr. of amputations:**  6 (8%) |
| **Waterworth 1975**    Retrospective analysis of autopsy reports    -    Civilians | **N**= 21    **Age:** -  **Sex:** -    **Multiple injuries:**  21 (100%)    **Mortality:**  Paper only reports fatalities  **KIA:** 18 (86%)  **DOW**: 3 (14%) | Birmingham, UK    **Time frame:**  November 21, 1974    **Cause:**  bomb | **Unknown** | **Nr. of injured persons:**   **Head/neck**  **Face**  **Thorax**  **Abdomen**  **Extremities**  **Spine**  **Pelvis**  **External**  **Burns** | 21 (100%)    15 (71%)  11 (52%)  18 (85%)  15 (67%)  14 (67%)  -  -  5 (24%)  19 (91%) | **Nr. of extremity injuries:**  31 (100%)    **Upper extremity injuries:**-    **Lower extremity injuries:**  31 (100%)    **Total nr. of amputations:**  15 (48%) |
| **Weil 2005**  Retrospective hospital data    Adults & children    Civilians | **N**= 44    **Age:**  Ø 20 (±12.8)  **Sex:** -    **Multiple injuries:**34 (77%)    **Mortality**  **KIA:** -  **DOW:** 3 (7%) | Israel    **Time frame:**  2000 - 2003    **Cause:**  Suicide bombing | **Unknown** | **Nr. of injured persons:**    **Head**  **Face**  **Thorax**  **Abdomen**  **Extremities**  **Spine**  **Pelvis**  **External**  **Burns** | 44 (100%)    -  -  -  -  44 (100%)  -  -  -  - | **Nr. of extremity injuries:**  66 (100%)    **Type IIIA-IIIB fractures:**  57 (86%)    **Type IIIc fractures:**  6 (9%)    **compartment syndrome:**  1 (2%)    **Total nr. of amputations:**  2 (3%) |
| **Wong 2006**    Case report    Adults    Civilian | N= 5    **Age:**  Ø 39,8 (27-53)  **Sex:** M 2 / F 3    **Multiple injured:**3 (60%)    **Mortality**  **KIA:** -  **DOW**: - | London, UK    **Time frame:**  July 7, 2005    **Cause:**  Suicide bombs on underground and bus | **Unknown** | **Nr. of injured persons**    **Head**  **Face**  **Thorax**  **Abdomen**  **Extremities**  **Spine**  **Pelvis**  **External**  **Burns** | 5 (100%)      -  1 (20%)  -  -  5 (100%)  1 (20%)  -  -  2 (40%) | **No. of extremity injuries:**  7 (100%)    **Upper extremity injuries:**  0 (0%)    **Lower extremity:** 7 (100%)    **Total nr. of amputations:**  4 (57%) |
| **Yasin 2011**  Retrospective hospital data    Adults    Civilians & military | **N**= 1,296    **Age:** Ø 33.4 (±2.3)  **Sex:** M 1,271/ F 25    **Multiple injuries:**  1,296 (100%)    **Mortality**  **KIA:** 95 (7%)  **DOW:** 91 (7%) | Pakistan    **Time frame:**  2008 - 2011    **Cause:**  Suicide bombing | **Unknown** | **Nr. of injuries:**    **Head**  **Face**  **Thorax**  **Abdomen**  **Extremities**  **Spine**  **Pelvis**  **External**  **Burns**  **urogenital injuries** | 5,767    143 (3%)  467 (8%)  1,105 (19%)  1,156 (20%)  853 (15%)  35 (0.6%)  -  1,917(33%)  -  91 (1.5%) | **Nr. of extremity injuries**  853 (100%)    **Upper extremity amputation:**  14 (1.6%)    **Lower extremity amputation:**  101 (11.8%) |
| **Yazgan 2016**    Retrospective hospital data    Adults    Civilian | N= 28    **Age:** Mdn 42 (range 18-65)  **Sex:** M 21 /F 7    **Multiple injured:** -    **Mortality**  **KIA:** -  **DOW:** 5 (16%) | Ankara, Turkey    **Time frame:**  July 7, 2005    **Cause:**  Suicide bomb | **Unknown** | **Nr of injured persons:**    **Head**  **Face**  **Thorax**  **Abdomen**  **Extremities**  **Spine**  **Pelvis**  **External**  **Burns** | 28 (100%)    6 (21%)  10 (35%)  3 (11%)  5 (18%)  6 (21%)  1 (4%)  -  11 (39%)  - | **No. of injuries:**  6 (100%) |

*Legend: DOW = died of wounds; F = Female; KIA = killed in action; M = male; N = sample size; nr. = number; y= years; Ø = not reported; x̄ = mean; ± = standard deviation*; *Color indicates the same data source: ITR; Oklahoma bombing; Madrid March 2004; Dharan June 1996; London July 2005*

## B) Focus on specific body parts

| **Body part** | **Study ID** | **Severity of injuries** | **Specifics of the Injuries**  **– nr. of injuries (%)** | |
| --- | --- | --- | --- | --- |
| **Chest injuries** | **Bala 2010** | **Unknown** | **Total nr. of chest injuries:**   - Cardiac laceration - Esophageal tears - Airway injury - Diaphragm injury - Diaphragmatic tear - Pneumo-/hemothorax - Lung contusion - Rib fracture | **64**  1 (2%)  1 (2%)  1 (2%)  1(2%)  1(2%)  22 (34%)  29 (45%)  8 (13%) |
| **Amputation** | **Patel 2012** | **Unknown** | **Total nr. of amputations**    **Nr. of traumatic upper-limb amputations**   - Shoulder amputations - Above-elbow amputation - Below-elbow amputation - Wrist amputation     **Nr. of traumatic lower-limb amputations**   - Above-knee amputations - Below-knee amputation - Through-knee amputations - Hip amputations | **91**    **19**  0 (0%)  6 (6%)  11 (12%)  2 (2%)      **72**  19 (21%)  44 (48%)  6 (6%)  3 (3%) |
| **Burn injuries** | **Chukwu-Lobelu 2017** | **Unknown** | **Pattern of burn injuries:**   - Survivable burn injuries (up to 50% total body surface area (TBSA)) - Potential survivable burn injuries (51-80% TBSA) - Non-survivable burn injuries (more than 80% TBSA) | **52**  28 (54%)    4 (8%)      20 (39%) |
|  | **Rosenberg 1982** | **Unknown** | **Nr. of patients with burn injuries**   - <30% TBSA - >30% TBSA | **12**  8 (67%)  4 (33%) |
| **Vascular injuries** | **Heldenberg 2016** | **Unknown** | **Nr. of injured blood vessels**  **Head/neck**   - Intracranial vessels - Carotid vessels - Others     **Thorax**   - Thoracic aorta - Innominate and subclavian vessels - Pulmonary artery - Others     **Abdomen & Pelvis**   - Inferior vena cava - Celiac and mesenteric arteries - Portal and splenic veins - Others     **Extremities**   - Axillary vessels - Brachial vessels - Ulnar and radial vessels - Other upper extremity - Femoral vessel - Popliteal vessel - Tibial vessel | **119**  **27 (23%)**  15  8  4      **15 (13%)**  4  7  3  1    **17 (14%)**  7  3  3  4      **60 (50%)**  6  8  5  1  20  10  10 |
| **Abdominal injuries** | **Bala 2008** | **Unknown** | **Nr. of abdominal injuries**   - Spleen - Liver - Small bowel - Large bowel - Stomach and duodenum - Urinary bladder - Diaphragm | **45**  3 (7%)  5 (11%)  10 (22%)  14 (31%)  6 (13%)  5(11%)  2 (5%) |
| **Maxillofacial injuries** | **Odhiambo 2002** | **Unknown** | **Nr. of Maxillofacial injuries**   - Ophthalmologic injuries - Cheeks/face - Forehead - Scalp - Lips - Dento-alveolar - Ears - Unspecific multiple injuries | **313**  80 (21%)  70 (18%)  54 (14%)  48 (12%)  19 (5%)  13 (3%)  10 (3%)  19 (5%) |
|  |  | **Unknown** | **Details ophthalmologic injuries**    **Nr. of ophthalmologic injuries**   - Cornea /scleral perforation - Cuts and lacerations of eyelids - Conjunctival haemorrhage - Foreign body - Ruptured eyeball - Unspecific | **80**  17 (21%)  18 (23%)  14 (18%)  6 (8%)  4 (5%)  21 (26%) |
|  | **Ahmad 2024** | **Unkown** | **Nr. of injuries:**  **Soft tissue injuries:**   - Bruises - Facial lacerations - Avulsions - Combinations   **Hard tissue injuries**   - Frontal bone - Midface - Mandible - Multiple facial | 207 (100%)  129 (62%)  25 (12%)  31 (15%)  19 (9%)  54 (26%)  78 (38%)  6 (3%)  23 (11%)  30 (14%)  19 (9%) |
| **Ear injuries** | **Leibovici 1999** | **Unknown** | Nr. of patients with isolated eardrum perforations | 142 (21.9%) |
| **Eye injuries** | **Mines 2000** | **Unknown** | **Nr. of patients with eye injuries**   - bilateral ocular injuries - unilateral ocular injuries - additional face fractures | **55**  12 (22%)  43 (78%)  13 (24%) |
|  | **Thach 2000** | **Unknown** | **Nr. of patients with eye injuries**   - bilateral corneoscleral laceration - unilateral corneoscleral laceration | **3**  1 (33%)  2 (67%) |

*Legend: nr. = number; TBSA = total burn surface area; ABSI = abbreviated burn severity index.*

*Color indicates the same data source: ITR; Oklahoma bombing; Madrid March 2004; Dharan June 1996; London July 2005*
